# Analysis of survival data with cure fraction and variable selection: A pseudo-observations approach

**DOI:** 10.1177/09622802221108579

**Published:** 2022-06-27

**Authors:** Chien-Lin Su, Sy Han Chiou, Feng-Chang Lin, Robert W Platt

**Affiliations:** 1 Department of Epidemiology, Biostatistics and Occupational Health, 5620 McGill University, Montréal, Québec, Canada; 2 Centre for Clinical Epidemiology, Lady Davis Institute, Jewish General Hospital, Montréal, Québec, Canada; 3 Peri and Post Approval Studies, Strategic and Scientific Affairs, PPD, part of Thermo Fisher Scientific, Montréal, Québec, Canada; 4 Department of Mathematical Sciences, University of Texas at Dallas, Richardson, TX, USA; 5 Department of Biostatistics, University of North Carolina, Chapel Hill, NC, USA

**Keywords:** Bounded cumulative hazard, Cox proportional hazard, high-dimensional covariates, mixture cure model, penalized generalized estimating equation

## Abstract

In biomedical studies, survival data with a cure fraction (the proportion of subjects cured of disease) are commonly encountered. The mixture cure and bounded cumulative hazard models are two main types of cure fraction models when analyzing survival data with long-term survivors. In this article, in the framework of the Cox proportional hazards mixture cure model and bounded cumulative hazard model, we propose several estimators utilizing pseudo-observations to assess the effects of covariates on the cure rate and the risk of having the event of interest for survival data with a cure fraction. A variable selection procedure is also presented based on the pseudo-observations using penalized generalized estimating equations for proportional hazards mixture cure and bounded cumulative hazard models. Extensive simulation studies are conducted to examine the proposed methods. The proposed technique is demonstrated through applications to a melanoma study and a dental data set with high-dimensional covariates.

## 1 Introduction

**Figure 1.**
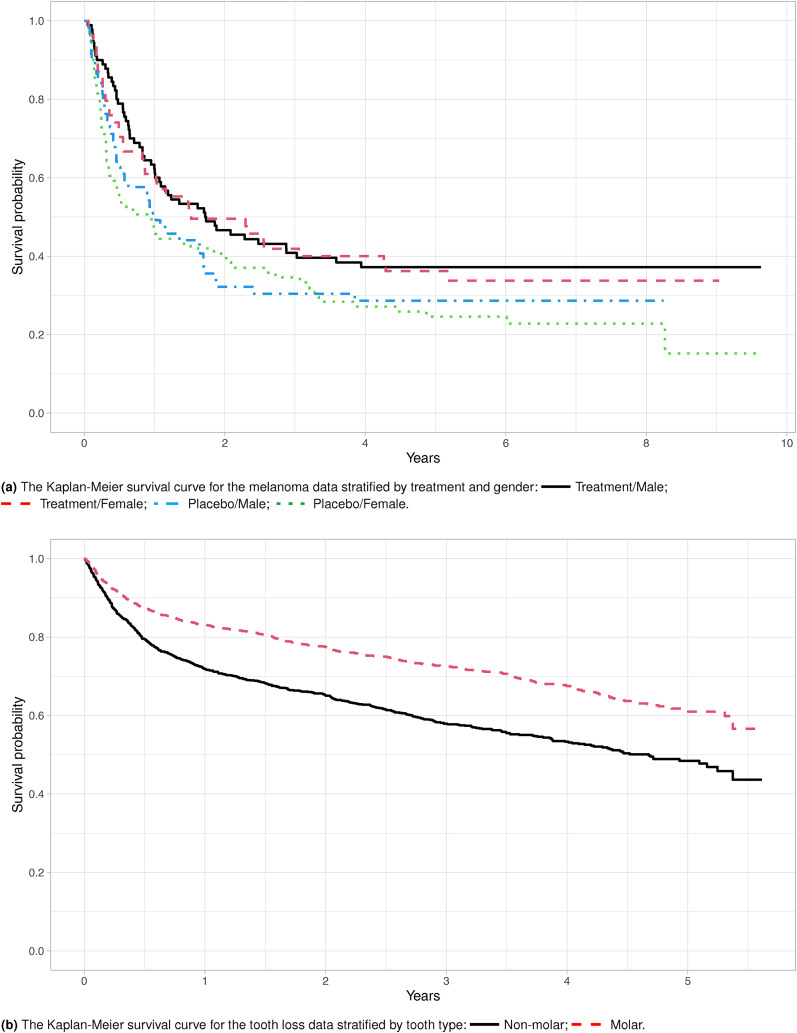
The Kaplan-Meier survival curves for the melanoma data and the tooth loss data to access potential cure fraction.

In the time-to-event analyzes, it is usually assumed that all subjects will eventually experience the event of interest if the follow-up period is sufficiently long. However, in many research fields, including biomedical, genetic, and social studies, some subjects may never experience the event of interest in their lifetime. These subjects are referred to as the cured or nonsusceptible subjects. For example, in a melanoma progression study,^1^ the cured patients never experience a melanoma relapse after the initial treatment. On the other hand, in a dental study,^2^ the cured subjects are the teeth that underwent proper periodontal treatments and can last a lifetime. In general, it is difficult to identify the cured subjects, but their presence is signaled by a leveling of the Kaplan-Meier (KM) survival curve at the end of the follow-up, for example, Figure 1(a). Standard models do not account for the cure fraction and could lead to biased estimates of the survival of susceptible subjects.^3^ Even if the cure fraction is accounted for, the dental study posts additional challenges on high-dimensionality. More than 50 predictors relevant to decision making in periodontal treatments are potential risk factors affecting the teeth’ survival. Motivated to tackle these emerging and challenging scientific questions, we propose a new estimating procedure using a pseudo-observations approach for the statistical inference on the parameters of interest and extend the proposed methods to regularized regression.

Two types of cure models have been popular in the literature, with most emphasis on the mixture cure (MC) model.^4^ The MC model assumes that the population consists of two types of patients, a cured group in which the patients are not at risk of experiencing the event and a susceptible group in which they eventually experience the event. The MC model consists of two components, *incidence* and *latency*. The former indicates whether the subject is susceptible, and the latter represents the time to the event when the patient is in the susceptible group. For the *incidence* component, a logistic regression model is often used to describe the covariate effects on the cure fraction. In contrast, parametric and semiparametric models have been proposed for the *latency* component to describe the underlying failure time distribution of susceptible subjects. Among those, Weibull model,^4^ generalized F model,^5^ Cox proportional hazards (PH) model,^6^ and accelerated failure time model^7^ have been studied.

The second type of cure model is the bounded cumulative hazard (BCH) model, also known as the promotion time cure model, which models the survival times through an improper survival function, e.g Tsodikov^8^ The idea was first introduced by Yakovlev et al.^9^ in biological considerations, in which cancer recurrence is promoted by the number of carcinogenic cells and disease progression. Thus, the parameters specified in the BCH model bear exact biological meaning. Treating the carcinogenic cell counts as latent and nuisance, the BCH model is suitable for any survival data types as long as it is reasonable to assume the data have a cure fraction.^10^ Incorporating covariates into the BCH model modifies the cure fraction and introduces a *long-term* covariate effect on the survival. The BCH model has a PH structure through the *long-term* effect parameter. Tsodikov et al.^11^ further incorporated covariates to the baseline survival function through another PH structure, introducing a *short-term* covariate effect on survival. The two-component BCH model of Tsodikov et al.^11^ is termed the PHPH model. The MC model and the PHPH model consist of different covariate effects, each providing unique clinical interpretations.

Existing estimating procedures for the MC model and the PHPH model usually involve updating the parameters via expectation-maximization (EM) algorithms to account for latent variables, for example, cure fraction, in the likelihood. However, these approaches could be computationally expensive in high-dimensional data or when the bootstrap method is used in variance estimation. Thus, estimating procedures such as the pseudo-observations approach that does not rely on the EM algorithm are more appealing for practical use. The concept of the pseudo-observations approach is to create pseudo values for the quantities of interest at individual levels using the analogy of leave-one-out cross-validation. These pseudo values are then treated as complete data where standard methods can be conveniently applied. Specifically, for subject i=1,…,n , let $T_{i}$ be independent and identically distributed random variables and $X_{i}$ be a vector of covariates. The interest lies in modeling $E[f(T_{i})|X_{i}]$ , where f is a pre-specified transformation function of $T_{i}$ . Due to censoring, not all $f(T_{i})$ are observed. However, the observed or unobserved $f(T_{i})$ can be replaced by its pseudo-observations ${\hat{\varrho }}^{i}=n\hat{\varrho }-(n-1){\hat{\varrho }}^{-i}$ where $\hat{\varrho }$ is a consistent and (approximately) unbiased estimator for the expectation ϱ=E[f(T)] and ${\hat{\varrho }}^{-i}$ is the estimator for ϱ using the remaining n−1 subjects, leaving subject i out from the sample. The pseudo-observations approach was first proposed by Andersen et al.^12^ to model the transition probabilities in multi-state models. Since then, the pseudo-observations approach has been applied to many settings in survival analysis, including survival estimates,^13^ the restricted mean survival times,^14^ the cumulative incidence function,^15^ the relative survival function,^16^ the illness-death model with interval-censored data,^17^ and the causal inference for recurrent event data.^18^ Large sample properties have also been thoroughly investigated.^19–21^ However, the pseudo-observations approach has not been applied to the analysis of survival data with a cure fraction.

Regularization and variable selection are commonly used in high-dimensional data analysis, but it has been less studied for cure models. Regularized procedures minimize an objective function that consists of a penalty function to reflect sparsity. Some of the popular penalty functions are the least absolute shrinkage and selection operator (LASSO),^22^ adaptive LASSO (ALASSO),^23^ and smoothly clipped absolute deviation (SCAD).^24^ In the Cox MC models, Liu et al.^25^ used LASSO and SCAD penalties to select variables based on penalized likelihood functions. Masud et al.^26^ performed the variable selection based on the ALASSO penalty while considering the linear and nonlinear effects in both components. Masud et al.^27^ further utilized the ALASSO penalty to the Cox MC and the BCH models. In both works, an EM algorithm was adopted to estimate the parameters, and the bootstrap resampling procedure was used to obtain standard error estimates. Their works can be computationally intensive in high-dimensional data. The penalized BCH model of Masud et al.^27^ has limited application as it requires additional information on the latent carcinogenic cell counts, making their approach not applicable for survival data without similar biological interpretations. Moreover, the *short-term* covariate effect is not considered in their approach, limiting the understanding of the covariate impact on the timing of disease occurrence.

In this article, we develop new estimating procedures based on the pseudo-observations approach for both the MC and the BCH models. We further extend the proposed method by adopting the penalized generalized estimating equations (PGEE) approach^28^ to perform variable selection. The proposed work closes the gap on variable selection in cure rate models with pseudo-observations techniques. The proposed approaches are attractive in several aspects. First, pseudo-observations can be straightforwardly used as complete outcomes for the generalized linear model (GLM) without indication of censoring. Second, the proposed estimating procedures are computationally efficient and faster in running time than standard approaches that adopt the EM algorithm for estimation and bootstrapping for standard errors as the unknown regression parameters are estimated via the generalized estimating equations (GEE) approach with corresponding variance estimates obtained by sandwich estimators. Third, unlike the estimation and variable selection of regression parameters in the *incidence* and *latency* component of the MC model^6,27^ or the *short-term* and *long-term* effects of the BCH model^11,27^ are performed simultaneously within one model, our proposed methods using pseudo-observations can perform the estimation and variable selection separately in each component of the MC model or each effect of the BCH model. Specifically, once the pseudo-observations for the quantity of interest for each component of the MC model or each effect of the BCH model are generated for each subject, they can be modeled with standard methods like GLMs. The GEE and PGEE estimating methods can be applied for parameter estimation and variable selection. Finally, the proposed regression estimators can be easily implemented via standard statistical software.

The remainder of the article is organized as follows. The MC model and the BCH model are reviewed in Section 2. The construction of pseudo-observations is described in Section 3. Inference procedure, model diagnosis, and variable selection are presented in Section 4. Comprehensive simulation results are reported in Section 5. The analysis of two real datasets is provided in Section 6. Concluding remarks are given in Section 7. Asymptotic properties and additional simulation results are provided in the online Supplemental Materials.

## 2 The cure models

Let Y denote the cure status of a subject, where Y=1 if the subject eventually experiences an event (uncured, susceptible), and Y=0 if the subject is a survivor (cured, non-susceptible). Let $T=YT^{*}+(1-Y)\times \infty$ be the survival time, where $T^{*}<\infty$ is the failure time if the subject is susceptible. In the presence of right censoring, we assume the observed data consist of n independent replicates $\left({\tilde{T}}_{i},\delta_{i},X_{i},Z_{i}\right)$ , i=1,…,n , which are copies of $\left(\tilde{T},\delta ,X,Z\right)$ , where $\tilde{T}=\min\{T,C\}$ , δ=I(T≤C) , C is the censoring time, and X and Z are vectors of covariates with dimensions p and q , respectively. We allow X and Z to be completely distinct, overlapped, or identical. When δ=1 , the subject experienced an event and Y=1 . However, when δ=0 , the cure status Y is not observed.

### 2.1 MC model

The MC model expresses the population survival function as

(1) $$S(t)=(1-\pi )+\pi S_{u}(t),$$

where π=P(Y=1) is the uncured rate and $S_{u}(t)$ is the conditional survival function of $T^{*}$ given Y=1 . The *incidence* component π is assumed to follow a logistic regression model

(2) $$\pi (X)=P(Y=1|X)=\frac{\exp\;(\alpha_{0}+\alpha^{⊤}X)}{1+\exp\;(\alpha_{0}+\alpha^{⊤}X)},$$

where $\alpha_{0}$ is a scalar and α is a p -column vector. For the *latency* component, we model the conditional survival function via the Cox proportional hazards model

(3) $$\lambda (t|Z)=\lambda_{0}(t)\exp\;(\beta^{⊤}Z),$$

where β is a q -column vector of regression coefficients, and $\lambda_{0}(t)$ is the unspecified baseline hazard function with the cumulative function $\Lambda_{0}(t)=\int_{0}^{t}\lambda_{0}(u)du$ . Under models (2) and (3), the MC model is called the PHMC model.^6^

### 2.2 BCH model

Suppose Λ(t) is the cumulative hazard function of $T^{*}$ such that Λ(∞)=θ>0 . Under the BCH model, the population survival function can be written as

(4) S(t)=exp{−θF(t)},

where F(t)=Λ(t)/θ is a proper cumulative distribution function of a nonnegative random variable with F(0)=0 and F(∞)=Λ(∞)/θ=1 . As t→∞ , one has $\lim_{t\to \infty }S(t)=\exp\;(-\theta )$ which indicates the cure rate. The covariate effects can be assumed on the impact of the parameter θ with $\theta (X)\equiv \theta (X,\gamma_{0},\gamma )$ , where $\theta (X,\gamma_{0},\gamma )$ is a known function that relates a p×1 vector of regression coefficients γ to X with an intercept term $\gamma_{0}$ . This modeling strategy leads to the improper PH model,^29^ and the covariates have a *long-term* effect because θ describes the long-term survival probability.^8^ A common choice of θ(⋅) is the exponential parameterization $\theta (X)=\exp\;(\gamma_{0}+\gamma^{⊤}X)$ . Tsodikov^8^ extends the improper PH model by adding a *short-term* effect by incorporating covariates into F(t) or survival function $\bar{F}(t)=1-F(t)$ . Specifically, the PHPH model of Tsodikov et al.^11^ has the form

(5) $$S(t)=\exp\;[-\theta (X)\{1-\bar{F}{\left(t\right)}^{\eta (Z)}\}],$$

where $\eta (Z)=\exp\;(\phi^{⊤}Z)$ and ϕ is a q -column vector of regression coefficients. To avoid overparameterization, we assume the coefficients ϕ do not contain an intercept term as suggested in Tsodikov et al.^11^When the cure fraction is the only parameter of interest, it makes no difference which model formulation (1) or (4) is chosen to estimate the cure rate nonparametrically. However, the two models become different when additional model assumptions are imposed on the cure rate and the latency distribution of $T^{*}$ in the MC model.

## 3 Pseudo-observations

### 3.1 Pseudo-observations for MC model

A common approach for constructing pseudo-observations is to generate those from a nonparametric estimator of the parameter of interest. The MC model has two parameters, uncured rate π and the conditional survival function $S_{u}(t)$ , to be estimated. There are two candidate estimators for the uncured rate π . The first one is proposed by Maller and Zhou,^30^ in which the cure rate 1−π was estimated by ${\hat{S}}_{\text{K}M}(t_{\max})$ , where ${\hat{S}}_{\text{K}M}(t)$ is the KM estimator^31^ and $t_{\max}$ is the maximum of the observed event times. The result implies that π can be estimated by ${\hat{\pi }}_{\text{K}M}=1-{\hat{S}}_{\text{K}M}(t_{\max})$ . Following the construction of pseudo-observations in Andersen et al.,^12^ one can define the pseudo-observations for subject i by

(6) $${\hat{\pi }}_{\text{K}M}^{i}=n\cdot {\hat{\pi }}_{\text{K}M}-(n-1)\cdot {\hat{\pi }}_{\text{K}M}^{-i},$$

where ${\hat{\pi }}_{\text{K}M}^{-i}=1-{\hat{S}}_{\text{K}M}^{-i}(t_{\max})$ is the estimator for π using the remaining n−1 subjects, leaving subject i out from the sample. The second estimator is based on the estimation of θ in Tsodikov^32^ through the connection between models (1) and (4). To be specific, let $t_{\left(1\right)}<t_{\left(2\right)}<\ldots <t_{\left(D\right)}$ be unique observed failure times, and let $t_{\left(0\right)}=0$ and $t_{\left(D+1\right)}=\infty$ . Let $M_{j}=\sum_{i=1}^{n}I({\tilde{T}}_{i}=t_{\left(j\right)},\delta_{i}=1)$ and $N_{j}=\sum_{i=1}^{n}I(t_{\left(j\right)}\leq {\tilde{T}}_{i}<t_{\left(j+1\right)},\delta_{i}=0)$ be the number of failure times at time $t_{\left(j\right)}$ and the number of censored times in the interval $\left[t_{\left(j\right)},t_{\left(j+1\right)}\right)$ , respectively. Under model (4), θ can be estimated by ${\hat{\theta }}_{\text{N}P}=\sum_{k=1}^{D}{\hat{\theta }}_{k}$ , where ${\hat{\theta }}_{k}=-\log\;\{(\sum_{\ell =k+1}^{D}M_{\ell }+\sum_{\ell =k}^{D}N_{\ell })/(\sum_{\ell =k}^{D}M_{\ell }+\sum_{\ell =k}^{D}N_{\ell })\}$ , k=1,…,D−1 , and ${\hat{\theta }}_{D}=-\log\;(N_{D}/(N_{D}+M_{D}))$ . Consequently, F(t) can be estimated by ${\hat{F}}_{\text{N}P}(t)=\sum_{\left\{j\;:\;t_{\left(j\right)}\leq t\right\}}{\hat{J}}_{\;j}$ , where ${\hat{J}}_{j}={\hat{\theta }}_{\;j}/{\hat{\theta }}_{\text{N}P}$ is the estimated jump size at time $t_{\left(j\right)}$ . Instead of using the Lagrange multiplier method,^32^ we use the change of variables approach under the condition $\sum_{k=1}^{D}J_{k}=1$ to obtain ${\hat{\theta }}_{\text{N}P}$ and ${\hat{F}}_{\text{N}P}(t)$ . The estimating procedure is summarized in Web Appendix A. Since models (1) and (4) have the same cure rate, one could estimate π by ${\hat{\pi }}_{\text{N}P}=1-\exp\;(-{\hat{\theta }}_{\text{N}P})$ and create the pseudo-observations for π by

(7) $${\hat{\pi }}_{\text{NP}}^{i}=n\cdot {\hat{\pi }}_{\text{NP}}-(n-1)\cdot {\hat{\pi }}_{\text{NP}}^{-i},$$

where ${\hat{\pi }}_{\text{N}P}^{-i}$ is the estimator of π obtained when leaving subject i out from the sample. Web Appendix B shows the behavior of pseudo-observations from (6) and (7) based on simulated data. One can see that these pseudo-observations are not necessary within the range [0,1].

To create the pseudo-observations for $S_{u}(t)$ , one can express $S_{u}(t)=\pi^{-1}\cdot (S(t)-(1-\pi ))$ from model (1) and imply that $S_{u}(t)$ can be estimated by ${\hat{S}}_{u,\text{K}M}(t)=\{{\hat{S}}_{\text{K}M}(t)-{\hat{S}}_{\text{K}M}(t_{\max})\}/\{1-{\hat{S}}_{\text{K}M}(t_{\max})\}$ . The pseudo-observations for $S_{u}(t)$ can then be created by

(8) $${\hat{S}}_{u}^{i}(t)=n\cdot {\hat{S}}_{u,\text{K}M}(t)-(n-1)\cdot {\hat{S}}_{u,\text{K}M}^{-i}(t),$$

where ${\hat{S}}_{u,\text{K}M}^{-i}(t)=\{{\hat{S}}_{\text{K}M}^{-i}(t)-{\hat{S}}_{\text{K}M}^{-i}(t_{\max})\}/\{1-{\hat{S}}_{\text{K}M}^{-i}(t_{\max})\}$ is the estimator for $S_{u}(t)$ when leaving subject i out from the sample.

### 3.2 Pseudo-observations for BCH model

Under model (4), we propose two approaches to create pseudo-observations for θ and F(t) , respectively. Since the cure rate $\lim_{t\to \infty }S(t)=\exp\;(-\theta )$ can be nonparametrically estimated by ${\hat{S}}_{\text{K}M}(t_{\max})$ , θ can be estimated by ${\hat{\theta }}_{\text{K}M}=-\log\;{\hat{S}}_{\text{K}M}(t_{\max})$ . Moreover, as mentioned in Section 3.1, θ can also be estimated by ${\hat{\theta }}_{\text{N}P}$ . Thus, the pseudo-observations for θ can be created by one of the following two approaches

(9) $${\hat{\theta }}_{\text{K}M}^{i}=n\cdot {\hat{\theta }}_{\text{K}M}-(n-1)\cdot {\hat{\theta }}_{\text{K}M}^{-i},$$

(10) $${\hat{\theta }}_{\text{NP}}^{i}=n\cdot {\hat{\theta }}_{\text{NP}}-(n-1)\cdot {\hat{\theta }}_{\text{NP}}^{-i}.$$

Based on Tsodikov,^8^ F(t) can be consistently estimated by ${\hat{F}}_{\text{K}M}(t)=\log\;({\hat{S}}_{\text{K}M}(t))/\log\;({\hat{S}}_{\text{K}M}(t_{\max}))$ . As mentioned in Section 3.1, F(t) can also be estimated by ${\hat{F}}_{\text{N}P}(t)=\sum_{\left\{j\;:\;t_{\left(j\right)}\leq t\right\}}{\hat{J}}_{\;j}$ . Thus, the pseudo-observations for F(t) can be created by one of the following two approaches,

(11) $${\hat{F}}_{\text{K}M}^{i}(t)=n\cdot {\hat{F}}_{\text{K}M}(t)-(n-1)\cdot {\hat{F}}_{\text{K}M}^{-i}(t),$$

(12) $${\hat{F}}_{\text{NP}}^{i}(t)=n\cdot {\hat{F}}_{\text{NP}}(t)-(n-1)\cdot {\hat{F}}_{\text{NP}}^{-i}(t),$$

where ${\hat{F}}_{\text{K}M}^{-i}(t)$ and ${\hat{F}}_{\text{N}P}^{-i}$ (t) are estimators of F(t) when leaving subject i out from the sample.

## 4 Statistical inference

The statistical inference of the pseudo-observations approach is based on the asymptotic unbiased property of the pseudo-observations for the parameter of interest.^33^ Following Jacobsen and Martinussen^20^ and Overgaard et al.,^21^ we present the proofs of the asymptotic unbiased property for proposed pseudo-observations in Web Appendix C of the online Supplemental Materials.

### 4.1 Estimation of parameters under MC model

Based on the pseudo-observations in Section 3.1, the parameters $\left(\alpha_{0},\alpha^{⊤}\right)$ in the *incidence* component and β in the *latency* component of the PHMC model with (2) and (3) can be estimated separately. To estimate $\left(\alpha_{0},\alpha^{⊤}\right)$ , we consider the following GLM

(13) $$g_{1}(E[Y_{i}|X_{i}])=\alpha_{0}+\alpha^{⊤}X_{i},$$

where $g_{1}(x)=\log\;\{x/(1-x)\}$ is the logit link function for a binary variable. The parameters $\left(\alpha_{0},\alpha \right)$ can be estimated based on the GEE approach^34^ using pseudo-observations ${\hat{\pi }}_{i},i=1,\ldots ,n$ by solving the estimating equations

(14) $$U(\alpha_{0},\alpha )=\sum_{i=1}^{n}\frac{\partial g_{1}^{-1}(\alpha_{0}+\alpha^{⊤}X_{i})}{\partial (\alpha_{0},\alpha )}V_{1,i}^{-1}({\hat{\pi }}^{i}-g_{1}^{-1}(\alpha_{0}+\alpha^{⊤}X_{i}))=0,$$

where ${\hat{\pi }}^{i}$ is the pseudo-observations for π , and $V_{1,i}$ is the working variance. Let $\left({\hat{\alpha }}_{0,\text{K}M}^{\text{P}O},{\hat{\alpha }}_{\text{K}M}^{\text{P}O}\right)$ and $\left({\hat{\alpha }}_{0,\text{N}P}^{\text{P}O},{\hat{\alpha }}_{\text{N}P}^{\text{P}O}\right)$ be the estimators from (14) as ${\hat{\pi }}^{i}$ is replaced by ${\hat{\pi }}_{\text{K}M}^{i}$ and ${\hat{\pi }}_{\text{N}P}^{i}$ , respectively.

To estimate β , the pseudo-observations for $S_{u}(t)$ are evaluated at several time points and used as responses in the GLM for the covariate effects. Specifically, let $t=\{t_{1},\ldots ,t_{H}\}$ be a set of distinct times between 0 and the maximum of observed event time, and let ${\hat{S}}_{u}^{i}(t_{h})$ be the pseudo-observations (8) for subject i at time $t_{h}$ for h=1,…,H . We assume the GLM with

(15) $$g_{2}(E[I(T_{i}^{*}>t_{h})|Z_{i}])=\xi_{t_{h}}+\beta^{⊤}Z_{i},$$

where $\xi_{t_{h}}$ is the intercept at time $t_{h}$ , β is the regression parameters, and $g_{2}(x)$ is a link function. Common choices for $g_{2}$ include the log-log function log{−log(x)} and log function log(x) . Model (15) becomes the Cox PH model (3) when $g_{2}(x)=\log\;\{-\log\;(x)\}$ and $\xi_{t_{h}}=\log\;\Lambda_{0}(t_{h})$ . We use the following GEE to estimate the unknown parameters β and $\xi_{H}=\{\xi_{t_{1}},\ldots ,\xi_{t_{H}}\}$

(16) $$U(\psi )=\sum_{i=1}^{n}\frac{\partial g_{2}^{-1}(t,\psi ;Z_{i})}{\partial \psi }V_{2,i}^{-1}({\hat{S}}_{u}^{i}(t)-g_{2}^{-1}(t,\psi ;Z_{i}))=0,$$

where $\psi =(\xi_{H},\beta )$ , ${\hat{S}}_{u}^{i}(t)=({\hat{S}}_{u}^{i}(t_{1}),\ldots ,{\hat{S}}_{u}^{i}(t_{H}){)}^{⊤}$ , $g_{2}^{-1}(t,\psi ;Z_{i})$ is a H×1 vector whose $j^{th}$ component equals $g_{2}^{-1}(\xi_{t_{j}}+\beta^{⊤}Z_{i})$ , and $V_{2,i}$ is a H×H working covariance matrix that accounts for the correlation inherent from the pseudo-observations.^12^We denote ${\hat{\psi }}^{\text{P}O}=({\hat{\xi }}_{H}^{\text{P}O},{\hat{\beta }}^{\text{P}O})$ as the estimators obtained from solving (16).

### 4.2 Estimation of parameters under BCH model

Under PHPH model (5), the *short-term* and *long-term* covariate effects can be estimated separately. To estimate the *long-term* effect $\left(\gamma_{0},\gamma \right)$ , we consider the following GLM

(17) $$g_{3}({\hat{\theta }}_{i})=\gamma_{0}+\gamma^{⊤}X_{i}+\varepsilon_{i},$$

where $\varepsilon_{i}$ , i=1,…,n , are independent and identically distributed (i.i.d.) with mean zero, and $g_{3}$ is a link function. Possible choices of $g_{3}(\cdot )$ are the log link function log(x) and the log-log function log{−log(x)} . Of these, setting $g_{3}(x)=\log\;(x)$ leads to the assumption $\theta (X_{i})=\exp\;(\gamma_{0}+\gamma^{⊤}X_{i})$ of model (5), which motivates us to estimate the parameters $\left(\gamma_{0},\gamma \right)$ based on pseudo-observations created by ${\hat{\theta }}_{\text{K}M}^{i}$ or ${\hat{\theta }}_{\text{N}P}^{i}$ via

(18) $$U(\gamma_{0},\gamma )=\sum_{i=1}^{n}\frac{\partial g_{3}^{-1}(\gamma_{0}+\gamma^{⊤}X_{i})}{\partial (\gamma_{0},\gamma )}V_{3,i}^{-1}({\hat{\theta }}^{i}-g_{3}^{-1}(\gamma_{0}+\gamma^{⊤}X_{i}))=0,$$

where ${\hat{\theta }}^{i}$ is the pseudo-observations for θ , and $V_{3,i}$ is a working variance of ${\hat{\theta }}_{i}$ . Let $\left({\hat{\gamma }}_{0,\text{K}M}^{\text{P}O},{\hat{\gamma }}_{\text{K}M}^{\text{P}O}\right)$ and $\left({\hat{\gamma }}_{0,\text{N}P}^{\text{P}O},{\hat{\gamma }}_{\text{N}P}^{\text{P}O}\right)$ be the estimators obtained from (18) when ${\hat{\theta }}_{i}$ is replaced by ${\hat{\theta }}_{\text{K}M}^{i}$ and ${\hat{\theta }}_{\text{N}P}^{i}$ in (9) and (10), respectively. Under the assumption $\eta (Z_{i})=\exp\;(\phi^{⊤}Z_{i})$ , we have $\log\;[-\log\;(1-F^{i}(t))]=\phi^{⊤}Z_{i}+\log\;[-\log\;(\bar{F}(t))]$ , where $F^{i}(t)=1-\bar{F}{\left(t\right)}^{\eta (Z_{i})}$ . We thus consider the GLM with

(19) $$g_{4}(1-{\hat{F}}^{i}(t_{h}))=\varsigma_{t_{h}}+\phi^{⊤}Z_{i}+\epsilon_{i},$$

where $\varsigma_{t_{h}}=\log\;\{-\log\;(\bar{F}(t_{h}))\}$ , $t_{h}\in t=\{t_{1},\ldots ,t_{H}\}$ , $g_{4}(x)=\log\;\{-\log\;(x)\}$ , and $\epsilon_{i}$ , i=1,…,n , are i.i.d. with mean zero. We consider the following GEE to estimate ϕ and $\varsigma_{H}$

(20) $$U(\Theta )=\sum_{i=1}^{n}\frac{\partial g_{4}^{-1}(t,\Theta ;Z_{i})}{\partial \Theta }V_{4,i}^{-1}((1-{\hat{F}}^{i}(t))-g_{4}^{-1}(t,\Theta ;Z_{i}))=0,$$

where $\Theta =(\varsigma_{H},\phi )$ , ${\hat{F}}^{i}(t)=({\hat{F}}^{i}(t_{1}),\ldots ,{\hat{F}}^{i}(t_{H}){)}^{⊤}$ is the vector of pseudo-observations for $F^{i}(t)$ calculated at $t=\{t_{1},\ldots ,t_{H}\}$ for subject i , $g_{4}^{-1}(t,\Theta ;Z_{i})$ is the H -column vector whose jth component equals $g_{4}^{-1}(\varsigma_{t_{j}}+\phi^{⊤}Z_{i})$ , and $V_{4,i}$ is a H×H working covariance matrix. Let ${\hat{\Theta }}_{\text{K}M}^{\text{P}O}=({\hat{\varsigma }}_{H,\text{K}M}^{\text{P}O},{\hat{\phi }}_{\text{K}M}^{\text{P}O})$ and ${\hat{\Theta }}_{\text{N}P}^{\text{P}O}=({\hat{\varsigma }}_{H,\text{N}P}^{\text{P}O},{\hat{\phi }}_{\text{N}P}^{\text{P}O})$ be the estimators obtained from equations (20) while ${\hat{F}}^{i}(t)$ is replaced by ${\hat{F}}_{\text{K}M}^{i}(t)$ and ${\hat{F}}_{\text{N}P}^{i}(t)$ in (11) and (12), respectively.

### 4.3 Variance estimation and model diagnosis

All estimators obtained from solving estimating equations mentioned in Sections 4.1 and 4.2 can be using the geese function in the R package *geepack*.^35^ We adopt the approximate jackknife variance estimates,^36^ which is available in the geese function. Note that adopting a sandwich estimator might lead to inconsistent and upward biased results for variance estimation; however, this has an insignificant impact in practical applications.^20^ We follow the idea of pseudo-residuals^33^ to assess the goodness-of-fit for (13) and (15) for the PHMC model. Define the pseudo-residuals $\left\{{\hat{\pi }}^{i}-g_{1}^{-1}({\hat{\alpha }}_{0}+{\hat{\alpha }}^{⊤}X_{i});i=1,\ldots ,n\right\}$ based on either ${\hat{\pi }}_{\text{K}M}^{i}$ or ${\hat{\pi }}_{\text{N}P}^{i}$ , and $\left\{{\hat{S}}_{u}^{i}(t)-g_{2}^{-1}({\hat{\xi }}_{t}^{\text{P}O}+{\hat{\beta }}^{{\text{P}O}^{⊤}}Z_{i});i=1,\ldots ,n\right\}$ calculated at a given time t∈t . If the model fits the data well, no trend should be perceptible when plotting residuals against a covariate. Similarly, we consider pseudo-residuals $\left\{{\hat{\theta }}_{\text{K}M}^{i}-g_{3}^{-1}({\hat{\gamma }}_{0,\text{K}M}^{\text{P}O}+{\hat{\gamma }}_{\text{K}M}^{{\text{P}O}^{⊤}}X_{i});i=1,\ldots ,n\right\}$ or $\left\{{\hat{\theta }}_{\text{N}P}^{i}-g_{3}^{-1}({\hat{\gamma }}_{0,\text{N}P}^{\text{P}O}+{\hat{\gamma }}_{\text{N}P}^{{\text{P}O}^{⊤}}X_{i});i=1,\ldots ,n\right\}$ for model (17) and the pseudo-residuals $\left\{(1-{\hat{F}}_{\text{K}M}^{i}(t))-g_{4}^{-1}({\hat{\varsigma }}_{t,\text{K}M}^{\text{P}O}+{\hat{\phi }}_{\text{K}M}^{{\text{P}O}^{⊤}}Z_{i});i=1,\ldots ,n\right\}$ or $\left\{(1-{\hat{F}}_{\text{N}P}^{i}(t))-g_{4}^{-1}({\hat{\varsigma }}_{t,\text{N}P}^{\text{P}O}+{\hat{\phi }}_{\text{N}P}^{{\text{P}O}^{⊤}}Z_{i});i=1,\ldots ,n\right\}$ at a given time t∈t for model (19). The idea of pseudo-residuals is illustrated in the melanoma data in Section 6.1.

### 4.4 Variable selection

The proposed pseudo-observations approach allows variable selection and parameter estimation to be simultaneously implemented in each component of the PHMC model and the PHPH model by penalizing the corresponding GEEs.^28^Specifically, for the *incidence* component of the PHMC model, we penalize the GEE in (14) by

(21) $$S(\alpha_{0},\alpha )=U(\alpha_{0},\alpha )-q_{\lambda_{1}}(|(\alpha )|)\circ \text{sign}(\alpha ),$$

where for some p×1 vectors u and v , $q_{\lambda_{1}}(u)=\{q_{\lambda_{1}}(|u_{1}|),\ldots ,q_{\lambda_{1}}(|u_{p}|){\}}^{⊤}$ is a vector of penalty functions for some tuning parameter $\lambda_{1}$ , $\mathrm{sign}\;(u)=\{\mathrm{sign}\;(u_{1}),\ldots ,\mathrm{sign}\;(u_{p}){\}}^{⊤}$ , sign(x)=I(x>0)−I(x<0) , and u∘v is the element-wise product of u and v . The intercept term $\alpha_{0}$ is not penalized and has been left out of the penalty term in (21). Similarly, we consider the following PGEE for ψ for the *latency* component of the PHMC model by extending (16)

(22) $$S(\psi )=U(\psi )-q_{\lambda_{2}}(|\beta |)\circ \text{sign}(\beta ),$$

where $\lambda_{2}$ is the tuning parameter, and the intercept term, $\xi_{H}$ , is left out of the penalty term.

For the *long-term* effect of the PHPH model, we extend (18) to the following PGEE

(23) $$S(\gamma_{0},\gamma )=U(\gamma_{0},\gamma )-q_{\lambda_{3}}(|\gamma |)\circ \text{sign}(\gamma ),$$

where $\lambda_{3}$ is the tuning parameter and the intercept term $\gamma_{0}$ is not penalized.

Lastly, for the *short-term* effect of the PHPH model, we penalize (20) through

(24) $$S(\Theta )=U(\Theta )-q_{\lambda_{4}}(|\phi |)\circ \text{sign}(\phi ),$$

where $\lambda_{4}$ is the tuning parameter and $\varsigma_{t_{H}}$ is not penalized. In the simulation studies and data analyzes, we illustrate the proposed procedure with the SCAD penalty^24^ and select the tuning parameters via five-fold cross-validation, where the data are randomly partitioned into five subsets of approximately equal sizes. For a given tuning parameter λ , we calculate the overall cross-validated prediction error $CV(\lambda )=|N^{\left(k\right)}{|}^{-1}\sum_{\;j\in N^{\left(k\right)}}m_{\;j}^{-1}\sum_{\ell =1}^{m_{j}}(PR({\hat{\vartheta }}^{(-k{)}^{⊤}}W_{\;j\ell }){)}^{2}$ where ${\hat{\vartheta }}^{\left(-k\right)}$ is the PGEE estimator based on data without the k th subset, $\left|N^{\left(k\right)}\right|$ is the size of the k th subset, $m_{j}$ is the number of pseudo-observations for subject j , and $PR({\hat{\vartheta }}^{(-k{)}^{⊤}}W_{\;j\ell })$ are the pseudo-residuals as defined in Section 4.3 with covariates $W_{\;j\ell }$ . We use the estimates obtained from the unpenalized GEEs as the initial values in the iteration of PGEEs and cross-validation.

## 5 Simulation

Simulation studies are conducted to assess the finite sample performance of proposed estimators. We first evaluate the performance under the PHMC model. Specifically, we generate the cure status according to the logistic model (2) with one covariate $X_{i},i=1,\ldots ,n$ , generated from a Bernoulli(0.5) distribution. The regression coefficient is set at $\alpha =(\alpha_{0},-1)$ so that the treatment group (e.g. those with $X_{i}=1$ ) are more likely to be cured. The intercept is chosen to be $\alpha_{0}=2.8$ , $\alpha_{0}=2$ or $\alpha_{0}=0.9$ to achieve the average cure rates of 10% (14.1% among those with $X_{i}=1$ ), 20% (26.7% among those with $X_{i}=1$ ) or 40% (52.3% among those with $X_{i}=1$ ), respectively. On the other hand, the survival times are generated from the Cox PH model (3), with $\lambda_{0}(t)=1/3$ , and $\left(Z_{i1},Z_{i2}\right)$ generated from independent Bernoulli(0.5) and Uniform(0, 1), respectively. We set the regression coefficient in (3) at β=(1,0.5) and generated the censoring times from Uniform(0, c ), where c is chosen to make 10% of the censored subjects susceptible. Throughout the simulations, the pseudo-observations are calculated at 10 time-points from the quantitles of observed event times between 0 and $t_{\max}$ .

Table 1 summarizes the simulation results of 20% and 40% cure rates scenarios based on (14) and (16) with link functions $g_{1}(x)=\log\;\{x/(1-x)\}$ , $g_{2}(x)=\log\;\{-\log\;(x)\}$ and $\xi_{t_{h}}=\log\;\Lambda_{0}(t_{h})$ . The scenario with 10% cure rate is presented in Table 1 in Web Appendix E. We only present the results with a working independence assumption among the pseudo-observations. Adopting a more complicated covariance structure provides no obvious improvement as presented in Klein and Andersen^37^ and Graw et al.^19^The proposed estimates are compared with the EM-algorithm estimators obtained from Peng and Dear^6^ with B=100 bootstrap samples for standard error estimation. For each estimator, we report the average bias (Bias), the empirical standard error (ESE), the average of the standard error estimator (SEE), and the empirical coverage rate (CR) of 95% confidence interval based on 500 replicates with sample size n=200,400,600 , and 1000 . Overall, the proposed estimators perform reasonably in all scenarios with Bias, ESE, and SEE all decreasing with increasing n while CRs are close to the nominal level of 0.95. The estimators $\left({\hat{\alpha }}_{0,\text{N}P}^{\text{P}O},{\hat{\alpha }}_{\text{N}P}^{\text{P}O}\right)$ and $\left({\hat{\alpha }}_{0,\text{K}M}^{\text{P}O},{\hat{\alpha }}_{\text{K}M}^{\text{P}O}\right)$ have similar performance, indicating that pseudo-observations constructed by (6) or (7) are both appropriate. Compared to the estimates of Peng and Dear,^6^ our estimators have higher ESE and SEE under various sample sizes, indicating a loss of efficiency. Specifically, based on our simulation settings, the smallest relative efficiency loss for the *latency* component is around 12% with n=1000 under the 10% cure rate scenario. The smallest relative efficiency loss for the *incidence* component is around 9% with n=1000 under the 40% cure rate scenario. This loss efficiency situation is likely due to a discrete approximation to the baseline hazard function with a continuous time scale, for example, 10 selected time-points for the latency component in our approach. Note that the latency component yields smaller ESE and SEE under 10% cure rate compared to 20% and 40% cure rates scenarios as more noncured subjects contribute to the estimation of β .

**Table 1.** Simulation summaries under the PHMC model based on 500 replicates.

		Incidence						Latency			
n		${\hat{\alpha }}_{0,\text{N}P}^{\text{P}O}$	${\hat{\alpha }}_{1,\text{N}P}^{\text{P}O}$	${\hat{\alpha }}_{0,\text{K}M}^{\text{P}O}$	${\hat{\alpha }}_{1,\text{K}M}^{\text{P}O}$	${\hat{\alpha }}_{0}^{\text{E}M}$	${\hat{\alpha }}_{1}^{\text{E}M}$	${\hat{\beta }}_{1}^{\text{P}O}$	${\hat{\beta }}_{2}^{\text{P}O}$	${\hat{\beta }}_{1}^{\text{E}M}$	${\hat{\beta }}_{2}^{\text{E}M}$
20% cure rate, 30% censoring rate											
200	Bias	0.040	−0.037	0.038	−0.034	0.048	−0.026	0.011	0.005	0.009	0.001
	ESE	0.431	0.507	0.423	0.503	0.396	0.468	0.226	0.379	0.209	0.321
	SEE	0.473	0.560	0.469	0.556	0.349	0.477	0.232	0.381	0.207	0.335
	CR	0.954	0.963	0.957	0.963	0.923	0.968	0.964	0.966	0.960	0.968
400	Bias	0.018	−0.027	0.019	−0.028	0.032	−0.036	0.016	0.022	0.013	0.017
	ESE	0.328	0.377	0.327	0.377	0.286	0.325	0.159	0.277	0.138	0.230
	SEE	0.323	0.387	0.324	0.387	0.271	0.351	0.166	0.272	0.142	0.232
	CR	0.956	0.962	0.956	0.960	0.930	0.958	0.960	0.964	0.958	0.960
600	Bias	0.014	−0.013	0.011	−0.012	0.021	−0.017	0.009	−0.013	0.007	−0.013
	ESE	0.260	0.300	0.254	0.299	0.215	0.251	0.124	0.233	0.109	0.190
	SEE	0.266	0.316	0.264	0.315	0.205	0.272	0.136	0.223	0.115	0.188
	CR	0.960	0.954	0.964	0.950	0.956	0.976	0.970	0.932	0.974	0.936
1000	Bias	−0.010	0.008	0.008	0.010	0.015	−0.014	−0.001	−0.004	−0.002	0.000
	ESE	0.216	0.236	0.217	0.246	0.164	0.188	0.093	0.172	0.086	0.143
	SEE	0.201	0.230	0.199	0.238	0.161	0.206	0.104	0.173	0.088	0.144
	CR	0.940	0.954	0.954	0.958	0.940	0.974	0.964	0.946	0.958	0.946
40% cure rate, 50% censoring rate											
200	Bias	−0.027	−0.001	−0.025	−0.001	−0.012	−0.008	0.011	−0.045	−0.008	−0.044
	ESE	0.334	0.425	0.335	0.425	0.288	0.363	0.311	0.586	0.279	0.404
	SEE	0.312	0.411	0.312	0.411	0.242	0.368	0.328	0.556	0.271	0.439
	CR	0.926	0.958	0.926	0.958	0.886	0.950	0.954	0.946	0.936	0.964
400	Bias	−0.018	−0.019	−0.019	−0.020	−0.021	−0.004	−0.010	−0.014	−0.019	−0.010
	ESE	0.290	0.339	0.298	0.351	0.192	0.234	0.228	0.492	0.192	0.299
	SEE	0.266	0.331	0.283	0.347	0.162	0.251	0.239	0.412	0.184	0.298
	CR	0.938	0.974	0.934	0.976	0.892	0.966	0.944	0.942	0.944	0.954
600	Bias	−0.018	0.015	−0.018	0.015	−0.018	0.007	−0.012	−0.032	−0.008	−0.008
	ESE	0.169	0.224	0.169	0.225	0.156	0.199	0.161	0.367	0.137	0.248
	SEE	0.173	0.227	0.172	0.227	0.153	0.202	0.185	0.316	0.147	0.237
	CR	0.940	0.956	0.940	0.960	0.940	0.950	0.965	0.945	0.962	0.932
1000	Bias	−0.015	0.016	−0.015	0.009	−0.010	0.002	−0.024	−0.023	−0.017	−0.010
	ESE	0.129	0.168	0.128	0.168	0.119	0.153	0.119	0.234	0.108	0.185
	SEE	0.128	0.169	0.129	0.170	0.120	0.154	0.134	0.229	0.111	0.182
	CR	0.942	0.945	0.942	0.952	0.940	0.956	0.958	0.938	0.940	0.942

Bias: bias of parameter estimator; ESE: the empirical standard error; SEE: average of the standard error estimator; CR: coverage rate of the 95% confidence interval; PHMC: proportional hazards mixture cure; $\left({\hat{\alpha }}_{0}^{\text{E}M},{\hat{\alpha }}_{1}^{\text{E}M}\right)$ and $\left({\hat{\beta }}_{1}^{\text{E}M},{\hat{\beta }}_{2}^{\text{E}M}\right)$ are EM-algorithm based estimators with standard errors are estimated based on B=100 bootstrap samples^6^ which can be implemented via the R package *smcure*.^38^

On the other hand, Table 2 in Web Appendix E reports the average computing time in seconds for each estimator under the 10% cure rate scenario based on 500 replicates. For each fixed n , our proposed estimators including variance estimation have smaller total computing times (summation of latency and incidence) than that of the EM-algorithm estimate^6^ using B=100 bootstrap samples for standard error estimation. Computing times increase as long as the sample size increases. Among our proposed estimators for the incidence component, the computing time for the KM estimator (6) is faster than that of the estimator (7). Note that all results of computing times are implemented in R and performed on a Linux machine with an Intel Core i7-8565U processor and 15.4  GB memory.

We next evaluate the performance of the proposed estimators under the PHPH model (5). For subject i , two independent covariates $X_{i1}$ and $X_{i2}$ are generated from Bernoulli(0.5) and standard normal distribution, respectively. We set $\theta (X_{i})=\exp\;(\gamma_{0}+\gamma_{1}X_{i1})$ , $\eta (Z_{i})=\exp\;(\phi_{1}X_{i1}+\phi_{2}X_{i2})$ and $\bar{F}(x)=\exp\;(-2x)$ . It is not straightforward to simulate survival time from the improper survival function of model (5) as it has a positive mass at ∞ . We thus utilize the connection between the MC model (1) and the PHPH model (5) to generate survival times. The data generation algorithm is summarized in Web Appendix D. We set $\gamma_{1}=-0.1$ , $\left(\phi_{1},\phi_{2})=(0.4,-0.3\right)$ with two different values of $\gamma_{0}$ , 0.5 and −0.05 , which correspond to 20% and 40% cure rate, respectively. The censoring times are independently generated from Uniform(0, c ), where c is chosen so that 10% of the censored subjects are susceptible. Tables 3 and 4 presented in Web Appendix E show the results of estimators $\left({\hat{\gamma }}_{0,\text{K}M}^{\text{P}O},{\hat{\gamma }}_{1,\text{K}M}^{\text{P}O}\right)$ and $\left({\hat{\gamma }}_{0,\text{N}P}^{\text{P}O},{\hat{\gamma }}_{1,\text{N}P}^{\text{P}O}\right)$ obtained from (18) and estimators $\left({\hat{\phi }}_{1,\text{N}P}^{\text{P}O},{\hat{\phi }}_{2,\text{N}P}^{\text{P}O}\right)$ and $\left({\hat{\phi }}_{1,\text{K}M}^{\text{P}O},{\hat{\phi }}_{2,\text{K}M}^{\text{P}O}\right)$ obtained from (20), respectively. A maximum likelihood estimator (MLE) is implemented for comparison. The proposed estimates are virtually unbiased. The SEE is reasonably close to the ESE, and the CR is close to the nominal level. The two constructions of the pseudo-observations yield a similar pattern, indicating that both approaches are feasible for constructing pseudo-observations. The proposed estimators have higher ESE and SEE than MLE under different sample sizes, indicating a loss of efficiency. For example, the relative efficiency losses are around 19% and 25% for *long-term* and *short-term* effects, respectively, under the scenario, n=1000 , 40% cure rate, and 50% censoring rate.

To study the performance of variable selection under the PHMC model, we consider the covariate $X=(X_{1},\ldots ,X_{20})$ in which $X_{1},X_{2}$ are independently generated from Uniform(0,1), $X_{3}$ and $X_{4}$ are independently generated from Bernoulli(0.5), and $X_{5},\ldots ,X_{20}$ are generated from a multivariate normal distribution with $E(X_{i})=0$ and $\mathrm{Cov}\;(X_{i},X_{j})=0.5^{\left|i-j\right|}$ , for i,j=5,…,20 . We set the regression coefficients at $\alpha_{0}=1.1$ , $\alpha =(0,1,-1.2,0,0,-0.9,0.8,0,0,\ldots ,0{)}^{⊤}$ , and $\beta =(-0.7,0,1,0,-0.5,0.8,0,0,0,\ldots ,0{)}^{⊤}$ , and the baseline hazard function $\lambda_{0}(t)=1/3$ . Those configurations yield a cure rate of 30%. The censoring times are independently generated from Uniform (0,c) , where c is chosen so that either 10% or 30% of the censored subjects are susceptible. For each simulated data set, we fit the GEE full model that considers all covariates, the GEE Oracle model that only includes covariates with nonzero coefficients, the proposed PGEE model with SCAD penalty, and the PHMC model with LASSO and ALASSO penalties proposed by Masud et al.^27^

Table 2 summarizes the results for variable selection under the PHMC model with n=200 and 600. Based on 200 replicates, the average mean square error (MSE), true positives (TP), and false positives (FP) are reported. The MSE is defined as $\sum_{\;j=1}^{200}\|{\hat{\alpha }}^{\;j}-\alpha {\|}^{2}/200$ for *incidence* component and $\sum_{\;j=1}^{200}\|{\hat{\beta }}^{\;j}-\beta {\|}^{2}/200$ for *latency* component, where ${\hat{\alpha }}^{\;j}$ and ${\hat{\beta }}^{\;j}$ are the estimators of α and β based on the j th generated dataset. The TP and FP are calculated as the average number of selected covariates that have actual nonzero and zero coefficients, respectively. Five-fold cross-validation is used to determine the tuning parameter. For the *incidence* component, the two pseudo-observations approaches yield similar performances, making them practically identical. For all scenarios, the MSE of proposed PGEE approaches is smaller than that of the full model but is larger than that of the oracle model. However, it becomes closer to the MSE of the oracle model as n increases. In addition, when the censoring rate is 40%, the proposed PGEE approaches behave closer to the oracle model, have TP closer to the number of nonzero covariates, and decreasing FP as n increases. On the contrary, the FP of the LASSO and ALASSO estimators do not seem to decrease with increasing n . For the *latency* component, we consider the proposed PGEE with three different correlation structures: independence, exchangeable, and AR(1). The results of PGEE with independent structure tend to have slightly higher TP and FP and lower MSE than that of the PGEE with exchangeable and AR(1) correlation structure. However, the performances are close when the sample size is large and with 40% censoring rate, indicating that little advantage gains while specifying complicated correlation structure among pseudo-observations for variable selection. When the censoring rate is 60%, the TP decreases and the MSE increases for all the presented methods. Compared to the results based on LASSO and ALASSO, our proposed PGEE performs reasonably in identifying important variables with n=600 . The results with n=400 and 1000 reveal a similar trend and are presented in Table 5 in Web Appendix E.

**Table 2.** Simulation summaries for variable selection on the *incidence* and *latency* component of the PHMC model with 30% cure rate.

		40% censoring rate			60% censoring rate		
n		MSE	TP	FP	MSE	TP	FP
**Incidence**							
200	Full.NP	7.44	–	–	10.96	–	–
	Full.KM	7.45	–	–	10.96	–	–
	Oracle.NP	1.66	4	0	4.54	4	0
	Oracle.KM	1.65	4	0	4.54	4	0
	SCAD.NP	5.23	2.77	7.40	6.21	1.49	4.87
	SCAD.KM	5.18	2.77	7.59	6.36	1.49	4.72
	LASSO	3.33	1.12	0.78	4.56	0.26	0.15
	ALASSO	3.23	1.26	0.69	4.54	0.23	0.19
600	Full.NP	1.39	–	–	4.96	–	–
	Full.KM	1.35	–	–	4.99	–	–
	Oracle.NP	0.39	4	0	3.57	4	0
	Oracle.KM	0.38	4	0	3.63	4	0
	SCAD.NP	0.68	3.87	0.42	4.64	0.98	0.63
	SCAD.KM	0.68	3.86	0.42	4.69	1.00	0.70
	LASSO	1.20	3.81	2.06	4.24	0.86	0.86
	ALASSO	1.17	3.80	1.88	4.23	0.93	0.66
**Latency**							
200	Full.indep	1.61	–	–	3.87	–	–
	Full.exch	2.39	–	–	4.44	–	–
	Full.ar1	2.23	–	–	4.78	–	–
	Oracle.indep	0.42	4	0	1.06	4	0
	Oracle.exch	0.41	4	0	1.80	4	0
	Oracle.ar1	0.41	4	0	1.30	4	0
	SCAD.indep	0.99	3.17	2.32	2.40	0.54	0.95
	SCAD.exch	1.08	2.86	0.75	2.40	0.31	0.35
	SCAD.ar1	1.04	2.98	0.86	2.51	0.28	0.20
	LASSO	1.38	2.26	1.70	2.26	0.34	0.36
	ALASSO	0.67	3.34	0.79	1.90	1.36	0.58
600	Full.indep	0.35	–	–	1.30	–	–
	Full.exch	0.41	–	–	2.17	–	–
	Full.ar1	0.37	–	–	1.36	–	–
	Oracle.indep	0.12	4	0	0.58	4	0
	Oracle.exch	0.13	4	0	0.71	4	0
	Oracle.ar1	0.12	4	0	0.61	4	0
	SCAD.indep	0.21	3.87	0.77	1.20	2.38	1.50
	SCAD.exch	0.24	3.82	0.18	1.54	1.89	0.86
	SCAD.ar1	0.24	3.83	0.16	1.23	1.72	0.38
	LASSO	0.31	3.94	2.64	1.48	2.35	1.43
	ALASSO	0.14	3.96	0.27	0.66	3.45	0.49

Full: model includes all covariates; Oracle: model only includes the covariates with nonzero coefficients; Acronyms that ends with .NP or .KM indicates pseudo-observations ${\hat{\pi }}_{\text{N}P}^{i}$ or ${\hat{\pi }}_{\text{K}M}^{i}$ , respectively; Acronyms that ends with .indep, .exch and .ar1 indicates independence, exchangeable and AR(1) correlation structure among pseudo-observations, respectively; LASSO: PHMC model with LASSO penalty; ALASSO: PHMC model with ALASSO penalty; MSE: the average estimated mean square error; TP: the average true positives; FP: the average false positives;PHMC: proportional hazards mixture cure.

To investigate variable selection performance under the PHPH model, we generate the covariate vector following the PHMC model’s configuration to specify the *short-term* and *long-term* effects. In this case, we set $\gamma_{0}=0.85$ , γ=(0,0,−0.9,0,0,−0.7,0,1,0,…,0) , ϕ=(−0.5,0,0.8,0,−0.7,0,0,0,0,…,0) , and the baseline function $\bar{F}(x)=\exp\;(-2x)$ , resulting in a cure rate of 30%. The censoring times are independently generated from Uniform (0,c) , where c is chosen so that either 10% or 30% of the censored subjects are susceptible. Tables 6 and 7 in Web Appendix E depict the simulation results for *long-term* and *short-term* effects based on different sample sizes. The definition of the MSE for the *long-term* and *short-term* effects is similar to that of the *incidence* and *latency* components mentioned in the above paragraph. We observe that the two constructions of pseudo-observations yield similar results. For each sample size n , the MSE of the PGEE is smaller than that of the full model but is larger than that of the oracle model. As n increases, the MSE of the PGEE decreases and is close to that of the Oracle model. Also, the TP increases and the FP decreases. However, as expected, the MSE increases and the TP decreases when the censoring rate increases to 60%. Finally, based on our simulation results, we observe minimal performance gains when specifying complicated correlations among pseudo-observations. With the same simulation specifications, we observe an improvement in FP and a loss in TP (results not shown here) when a one-standard error rule is used to select the optimum tuning parameter.

## 6 Data analysis

### 6.1 The melanoma data

We apply the proposed method to a melanoma dataset from the Eastern Cooperative Oncology Group phase III clinical trial e1684,^1^ which is available from the R package *smcure*.^38^ The primary objective is to determine whether the high dose interferon alpha-2b (IFN) regimen in postoperative adjuvant therapy would lead to a significantly prolonged interval of relapse-free for melanoma. The event of interest is the relapse of melanoma. The interested covariates include treatment (0 = placebo, 1 = IFN), gender (0 = male, 1 = female), and age (centered to zero). After excluding missing data, a total of 284 subjects is included in the analysis. The overall censoring rate is 30.9%. Figure 1(a) shows the KM estimates stratified by treatment and gender. The KM estimates level off at the end of the study, suggesting a fraction of nonsusceptibility to the recurrence of melanoma. This observation is confirmed by the Maller-Zhou test^30^ with a p -value of <0.001 . Based on the KM curves, a male has a higher cure rate than a female in the treatment group, whereas it is reversed in the control group, implying an interaction between the treatment and gender. Thus, the interaction term is also considered in the data analysis.

The top panel of Table 3 presents the results from the PHMC model obtained by (14) and (16) with $g_{1}(x)=\log\;\{x/(1-x)\}$ , $g_{2}(x)=\log\;\{-\log\;(x)\}$ , and $\xi_{t_{h}}=\log\;\Lambda_{0}(t_{h})$ . The lower panel of Table 3 presents the results from the PHPH model obtained by (18) and (20) with $g_{3}(x)=\log\;(x)$ , $g_{4}(x)=\log\;\{-\log\;(x)\}$ , and $\varsigma_{t_{h}}=\log\;\{-\log\;(\bar{F}(t_{h}))\}$ . For comparison, we included the estimator^6^ based on the EM-algorithm with standard errors obtained from 500 bootstrapped samples. The two constructions ${\hat{\pi }}_{\text{K}M}^{i}$ and ${\hat{\pi }}_{\text{N}P}^{i}$ of the pseudo-observations for π yield similar patterns. For the *incidence* component, our proposed estimates ${\hat{\alpha }}_{\text{N}P}^{\text{P}O}$ and ${\hat{\alpha }}_{\text{K}M}^{\text{P}O}$ are similar to the estimate ${\hat{\alpha }}^{\text{E}M}$ . The treatment has a significantly adverse effect on the susceptibility (noncured), which means the treatment substantially improves the relapse-free (cured) rate, especially for males. The positive age effect indicates that older patients tend to have a higher relapse rate of melanoma. In the *latency* component, none of the four covariates are significantly associated with the failure time if patients are susceptible. However, female patients tend to have a lower risk of recurring melanoma than male patients in the treatment group but higher in the control group.

**Table 3.** Parameter estimates for the melanoma data.

**PHMC model**									
**Incidence**	${\hat{\alpha }}^{\text{E}M}$			${\hat{\alpha }}_{\text{N}P}^{\text{P}O}$			${\hat{\alpha }}_{\text{K}M}^{\text{P}O}$		
	Est.	SEE	p -value	Est.	SEE	p -value	Est.	SEE	p -value
Intercept	1.502	0.366	<0.001	1.671	0.563	0.003	1.737	0.584	0.003
Treatment	−0.866	0.426	0.042	−1.272	0.612	0.037	−1.294	0.633	0.040
Gender	−0.442	0.492	0.368	−0.614	0.614	0.315	−0.628	0.635	0.320
Age	0.018	0.014	0.189	0.024	0.013	0.066	0.024	0.013	0.066
Treatment:Gender	0.648	0.665	0.329	0.886	0.734	0.225	0.903	0.755	0.228

**Latency**	${\hat{\beta }}^{\text{E}M}$			${\hat{\beta }}^{\text{P}O}$		
	Est.	SEE	p -value	Est.	SEE	p -value
Treatment	−0.012	0.236	0.958	−0.009	0.299	0.975
Gender	0.266	0.258	0.303	0.151	0.310	0.629
Age	−0.007	0.007	0.266	−0.009	0.007	0.206
Treatment: Gender	−0.334	0.369	0.365	−0.151	0.383	0.696

**PHPH model**						
**Long-term effect**	${\hat{\gamma }}_{\text{N}P}^{\text{P}O}$			${\hat{\gamma }}_{\text{K}M}^{\text{P}O}$		
	Est.	SEE	p -value	Est.	SEE	p -value
Intercept	0.519	0.167	0.002	0.538	0.164	0.001
Treatment	−0.689	0.275	0.012	−0.668	0.266	0.012
Gender	−0.266	0.223	0.229	−0.259	0.218	0.231
Age	0.012	0.006	0.044	0.012	0.006	0.044
Treatment: Gender	0.467	0.390	0.228	0.453	0.378	0.227

**Short-term effect**	${\hat{\phi }}_{\text{N}P}^{\text{P}O}$			${\hat{\phi }}_{\text{K}M}^{\text{P}O}$		
	Est.	SEE	p -value	Est.	SEE	p -value
Treatment	0.586	0.500	0.247	0.565	0.487	0.252
Gender	0.557	0.482	0.253	0.537	0.469	0.258
Age	−0.017	0.010	0.104	−0.017	0.010	0.101
Treatment: Gender	−0.718	0.572	0.214	−0.689	0.557	0.220

Est.: Parameter estimate; SEE: Standard error estimate; ${\hat{\alpha }}^{\text{E}M}$ and ${\hat{\beta }}^{\text{E}M}$ are EM-algorithm estimators with standard errors are estimated based on 500 bootstrap samples obtained from the R package smcure.^38^

Under the PHPH model, the treatment has a significantly negative effect on the *long-term* effect, indicating that high dose IFN regimen increase the rate of relapse-free melanoma, especially for males. Older patients have a high possibility of recurring melanoma even though the effect is not statistically significant at the 5% nominal level. Those results are consistent with the findings in the *incidence* of the PHMC model. None of the four covariates are statistically significant at the 5% nominal level related to the *short-term* effect. The estimates indicate females are likely to have a more rapidly developing melanoma within the treatment group. However, the result is reversed in the control group, i.e., males are expected to experience melanoma sooner than females. Those findings are verified in Figure 1(a), where the KM estimate drops relatively faster for the females than for the males in the treatment group. In contrast, the KM estimates drop faster for the males than females in the control group.

To understand the covariate effect of cure rates in two models, we calculate the estimated cure rates in each group while holding the age variable at the average. The results presented in Table 6 of Web Appendix E suggests that there are higher cure rates among the treatment groups, echoing our finding that the treatment substantially improves the relapse-free (cured) rate. The estimated cure rates based on ${\hat{\alpha }}_{\text{N}P}^{\text{P}O}$ and ${\hat{\alpha }}_{\text{K}M}^{\text{P}O}$ under the PHMC model are close to that based on ${\hat{S}}_{\text{K}M}(t_{\max})$ ; however, the results based on ${\hat{\gamma }}_{\text{N}P}^{\text{P}O}$ and ${\hat{\gamma }}_{\text{K}M}^{\text{P}O}$ under the PHPH model tend to have higher cure rate except for the female in the treatment group. This suggests that the PHMC model is more conservative in estimating cure rates. To assess the goodness of fit, Figure 2 in Web Appendix F presents the boxplots of pseudo-residuals for models (13) and (15) of the PHMC model. The top panel shows the boxplots of pseudo-residuals stratified by treatment and gender based on the pseudo-observations ${\hat{\pi }}_{\text{K}M}^{i}$ from (6) and its corresponding estimators. The pseudo-residuals fluctuate around zero, indicating the adequacy of the proposed GLM even though the pseudo-residuals have a larger variation in the Control/Male group. The bottom panel illustrates the boxplots of pseudo-residuals based on pseudo-observations ${\hat{S}}_{u}^{i}(t)$ calculated at four given time points chosen from the quantiles of observed event times. The residuals are symmetric around 0 at any given time point, which implies the proposed GLM model fits the data well even though the variation of the pseudo-observations increases in the Control/Male group as the time increases. The boxplots of pseudo-residuals stratified by treatment and gender based on GLMs (17) and (19) under the PHPH model are also presented in Figure 3 of Web Appendix F. The top panel shows the residuals calculated based on pseudo-observations ${\hat{\theta }}_{\text{K}M}^{i}$ and its resulting estimators, and the bottom panel presents the pseudo-residuals calculated based on pseudo-observations ${\hat{F}}_{\text{K}M}^{i}(t)$ at four given time points. Compared to Figure 2 in Web Appendix F, the pseudo-residuals under the PHPH model tend to have more considerable variations. This result might suggest that the PHMC model fits the data better than the PHPH model.

### 6.2 The dental data

We apply the proposed PGEE approaches to a dental dataset from the Creighton University School of Dentistry.^2^The dataset contains dental records from 5336 patients with periodontal disease collected between August 2007 and March 2013. In this work, the outcome of interest is the time to the first tooth loss due to periodontal reasons for each patient, yielding a censoring rate of 74.1%. The data analysis includes a total of 44 risk factors, whose detailed descriptions can be found in Tables 3 and 4 in Calhoun et al.^2^ The length of the follow-up was 5.7 years, and the last event occurred at 5.37 years for both molar and non-molar groups. There were 35 and 20 teeth censored between the last event and the end of the study for the malor group and non-molars group, respectively. Figure 1(b) shows the KM survival curves stratified by the tooth type (3456 molars vs. 1880 non-molars) leveling off to nonzero probabilities, indicating a possible presence of a cured fraction in the population. This is also confirmed by the Maller-Zhou test^30^ with a p -value of <0.001 .

As the study aims to identify which factors are associated with tooth loss, the proposed PGEE provides a logical tool for risk factor screening. We perform the variable selection procedure in both the PHMC model and the PHPH model. Under the PHMC model, we apply the proposed PGEEs in (21) and (22) with pseudo-observations created by ${\hat{\pi }}_{\text{K}M}^{i}$ and ${\hat{S}}_{u}^{i}(t)$ , respectively. While under the PHPH model, we apply the proposed PGEEs in (23) and (24) with pseudo-observations created by ${\hat{\theta }}_{\text{K}M}^{i}$ and ${\hat{F}}_{\text{K}M}^{i}(t)$ , respectively. In both the PHMC model and the PHPH model, a working independence correlation is incorporated for the ten pseudo-observations obtained from the quantitles of observed event times. The tuning parameters are selected via five-fold cross-validation. For comparison, we also fit the PHMC model with LASSO and ALASSO penalties proposed by Masud et al.^27^ implemented in the R package *intsurv*.^39^In the variable selection procedure, the molar variable’s coefficient is not penalized since the variable effect is the research of interest. Tables 4 and 5 present the variable selection results. Under the PHMC assumption, when predicting whether a tooth is cured, the LASSO and ALASSO selected more variables as we observed in the simulation studies; see Table 2 and Table 5 in Web Appendix E. The common selected factors based on presented methods include mobility score (mobil), bleeding on probing (bleed), periodontal probing depth (pocket_mean), decayed surfaces new (decayed_new), decayed surfaces recurrent (decayed_recurrent), endodontic therapy (endo), filled tooth (filled_tooth) and recurrent decayed surfaces (decay_recur_sum). Only the variable filled tooth increases the chance of cure while others decrease the chance of cure. In addition, for the *latency*, the PGEE tends to identify more risk factors in predicting tooth survival time. The mobility score (mobil), decayed surfaces new (decayed_new) and filled tooth (filled_tooth) are three common selected factors related to the tooth survival time based on PGEE and LASSO. Under the PHPH model assumption, our proposed PGEE approach suggests that factors plaque score (plaque), bleeding on probing (bleed_ave), plaque index (plaque_ave), filled surfaces (filled_sum), recurrent decayed surfaces (decay_recur_sum), decayed and filled surfaces (dfs_sum), number of decayed teeth (decayed_tooth_sum) and age at baseline (baseline_age) are importantly associated with the *long-term* effect. Moreover, 20 factors are identified in association with the *short-term* effect, indicating losing the tooth more rapidly. Interestingly, we observe that 15 variables are both selected by the PGEE in the *latency* component of the PHMC model and the *short-term* effect of the PHPH model even though covariates are interpreted differently in the two models.

**Table 4.** Estimated coefficients based on different penalization approaches for the dental dataset.

	PHMC						PHPH	
	LASSO		ALASSO		PGEE		PGEE	
	Incidence	Latency	Incidence	Latency	Incidence	Latency	Long-term	Short-term
**Tooth-level factors**								
molar	−0.327	−0.268	−0.392	−0.224	−0.618	−0.063	−1.303	−0.048
mobil	0.541	0.328	0.718		1.125	0.751		0.671
bleed	0.003		0.002		0.002			−0.003
plaque						0.002	0.013	0.002
pocket_mean	0.428		0.417		0.746			
pocket_max								
cal_mean								
cal_max	0.063		0.066					
fgm_mean								
fgm_max								
filled					1.372			
decay_new	0.063	0.111	0.119		1.591	0.815		0.744
decay_recurrent	0.194		0.165		1.619	0.590		0.547
dfs					−1.295			
crown	0.020							
endo	1.091		1.088		2.074			−0.683
filled tooth	−0.431	−0.066	−0.450		−0.690	−0.859		−0.842
decayed tooth	0.524		0.577			−1.434		−1.522

**Table 5.** (Continuation of Table 4) Estimated coefficients based on different penalization approaches for the dental dataset.

	PHMC						PHPH	
	LASSO		ALASSO		PGEE		PGEE	
	Incidence	Latency	Incidence	Latency	Incidence	Latency	Long-term	Short-term
**Subject-level factors**								
bleed_ave						0.006	−0.019	0.006
plaque_ave						−0.007	0.041	−0.007
pocket_mean_ave	0.013		0.021					
pocket_max_ave	0.017		0.023					
cal_mean_ave								
cal_max_ave								
fgm_mean_ave								
fgm_max_ave								
filled_sum						−0.007	0.011	−0.001
filled_ave								
decay_new_sum			0.016			0.005		
decay_new_ave	0.788			0.493	1.286			
decay_recur_sum	0.097		0.087		0.427	−0.009	0.015	−0.028
decayed_recur_ave					−3.686			
dfs_sum						−0.002	0.033	−0.006
dfs_ave								
filled_tooth_sum						0.033		0.033
filled_tooth_ave								
decayed_tooth_sum	0.044		0.049				0.034	
decayed_tooth_ave					−0.981	0.619		0.641
missing_tooth_sum								
missing_tooth_ave					−1.683	0.597		0.988
total_tooth	0.002							
**Demographic factors**								
age at baseline	0.013		0.013			0.005	0.009	0.005
gender	−0.051		−0.032					
**Health factors**								
diabetes						0.589		0.654
Tobacco use	0.171		0.159					0.362

## 7 Conclusions

This article extends the pseudo-observations approach to the context of right-censored survival data with a cure fraction for two popular cure models, the MC model and the BCH model. Several estimators for the regression parameters related to the cure rate and the risk of experiencing the event are proposed under the PHMC model and the PHPH model. The proposed methods allow researchers to estimate the covariate effects separately and identify essential factors associated with the cure rate and the risk of failure. Simulation studies show that the proposed methods perform reasonably under finite sample sizes. We also demonstrate the proposed methodology on two real applications involving survival data with a cure fraction.

In this work, for the MC model, we propose two different incidence regression estimators ${\hat{\alpha }}_{\text{K}M}^{\text{P}O}$ and ${\hat{\alpha }}_{\text{N}P}^{\text{P}O}$ . For the BCH model, we consider two different regression estimators ${\hat{\gamma }}_{\text{K}M}^{\text{P}O}$ and ${\hat{\gamma }}_{\text{N}P}^{\text{P}O}$ for *long-term* effects and two different regression estimators ${\hat{\phi }}_{\text{K}M}^{\text{P}O}$ and ${\hat{\phi }}_{\text{N}P}^{\text{P}O}$ for *short-term* effects. In the simulation studies, each of the pairs of estimators performs similarly. However, from the computing time point of view, we recommend that researchers consider estimators based on KM estimator as its computing times is fast. On the other hand, as we showed in the simulation studies, our proposed estimators based on pseudo-observations under both MC and BCH models have larger ESE and SEE than those from the usual MC and BCH models. This efficiency loss is commonly seen in the pseudo-observations literature.^12,40^ As pointed out by a referee, the efficiency loss is a limitation for our proposed pseudo-observations approach on the cure models when the number of covariates is small, like our real data analysis in Section 6.1. However, modeling pseudo-observations constitutes a general and straightforward approach to simplify survival analysis. In our applications, the pseudo-observations approach brings several advantages. First, it is more flexible and feasible for parameter estimation and variable selection when the number of covariates is large. The estimating procedure can be performed separately for each component in the MC model and each effect in the BCH model. Second, the computing time based on the pseudo-observations approach is faster than standard approaches that use the EM algorithm for estimation and bootstrapping for standard errors. Finally, pseudo-residuals can be applied for model diagnosis via residual plots.

Our PHPH model links the *short-term* effect to the baseline survival function via a proportional hazard model. The proposed PHPH model can be extended to accommodate non-proportional hazard assumptions by considering an exponential transformation or an accelerated failure time model in the *short-term* effect.^8^On the other hand, we applied the pseudo-observations approach to the PHMC model. Another commonly used MC model is the accelerated failure time MC (AFTMC) model,^7^ in which the logistic regression model is considered to model the cure status in the incidence component and the AFT model, $\log\;(T)=X^{⊤}\beta +\varepsilon$ , is used to model the conditional survival function in the latency component, where ε is the error term with survival distribution $S_{\varepsilon }(\cdot )$ . Under AFTMC model, our proposed pseudo-observations (6) and (7) using GEE approach can be directly applied to estimate the unknown parameters in the incidence component. For the latency component, we discuss the feasibility of using the pseudo-observations approach to estimate β under AFT model in two folds. First, when the survival distribution $S_{\varepsilon }(\cdot )$ is known; that is, a parametric AFT model, one can write $S_{\varepsilon }^{-1}(S_{u}(t))=\log\;(t)-X^{⊤}\beta$ and treat it as a GLM with link function $S_{\varepsilon }^{-1}(\cdot )$ . To estimate β , one can create the pseudo-observations for $S_{u}(t)$ with given time points $t\in \{t_{1},\ldots ,t_{H}\}$ based on KM estimator as we proposed in equation (8) and then the GEE approach can be applied to obtain the estimates for β . However, one might need to program the estimating equation on their own because the geese function in the R package *geepack*^35^ only provides commonly seen link functions. Second, when the survival distribution $S_{\varepsilon }(\cdot )$ is unknown in a semi-parametric AFT model, it may not be straightforward to use the pseudo-observations approach to estimate β even though the pseudo-observations for $S_{u}(t)$ can be created. The main issue is the unknown survival function $S_{\varepsilon }(\cdot )$ , so is the inverse function $S_{\varepsilon }^{-1}(\cdot )$ . Further investigation is required and will be an interesting topic for future research.

We note some limitations of our proposed work for variable selection. Based on our simulation studies, the TP rate seems to be low for the incidence component of the MC model when n=200 . This results might be induced because only one time point is used to create the pseudo-observations for the cure rate. When applying our proposed approach to a small sample sizes like 200 in our simulation, researchers can investigate the stability of variable selection via the bootstrap approach proposed by Royston and Sauerbrei.^41^ In this work, we only report the selected coefficient estimates from the penalized approach for the dental data analysis. In practice, one might adopt a two-stage approach in which the inference is based on the selected model to obtain standard errors.^27^The validity of inferences based on the penalization and the goodness-of-fit assessments will be studied in future work.

## Supplemental Material

sj-pdf-1-smm-10.1177_09622802221108579 - Supplemental material for Analysis of survival data with cure fraction and variable selection: A pseudo-observations approach Supplemental material, sj-pdf-1-smm-10.1177_09622802221108579 for Analysis of survival data with cure fraction and variable selection: A pseudo-observations approach by Chien-Lin Su, Sy Han Chiou, Feng-Chang Lin and Robert W Platt in Statistical Methods in Medical Research
