# Supplementary material for: Analysis of survival data with cure fraction and variable selection: A pseudo-observations approach
Source: Stat Methods Med Res. 2022 Jun 27;31(11):2037–53. doi: 10.1177/09622802221108579 (PMC9660265; doi:10.1177/09622802221108579)
Supplement: sj-pdf-1-smm-10.1177_09622802221108579 - Supplemental material for Analysis of survival data with cure fraction and variable selection: A pseudo-observations approach [file sj-pdf-1-smm-10.1177_09622802221108579.pdf]

Web-based Supplementary Materials for 'Analysis of survival data with cure fraction and variable selection: A pseudo-observations approach'

Chien-Lin Su[1,2,3], Sy Han Chiou[4], Feng-Chang Lin[5], and Robert W. Platt[1,2]

[1]Department of Epidemiology, Biostatistics and Occupational Health, McGill University, Montréal, Québec, Canada
[2]Centre for Clinical Epidemiology, Lady Davis Institute, Jewish General Hospital, Montréal, Québec, Canada
[3]Peri and Post Approval Studies, Strategic and Scientific Affairs, PPD, part of Thermo Fisher Scientific, Montréal, Québec, Canada
[4] Department of Mathematical Sciences, University of Texas at Dallas, Richardson, Texas
[5]Department of Biostatistics, University of North Carolina, Chapel Hill, NC, USA
chien-lin.su@mail.mcgill.ca

In this online supplementary materials, we provide the details that have been left out of the main paper. The R codes with examples for this work are deposited to a Github repository https://github.com/stc04003/pseudo-cure.

Web Appendix A

In this Appendix, we summarize the estimating procedures for obtaining nonparametric estimators $\hat{\theta}_{\text{NP}}$ and $\hat{F}_{\text{NP}}(t)$ proposed by Tsodikov [1]. Let $t_{(1)} < t_{(2)} < ... < t_{(D)}$ be observed failure time points with $t_{(0)} = 0$ and $t_{(D+1)} = \infty$. Denote $M_j = \sum_{i=1}^n I(\tilde{T}_i = t_{(j)}, \delta_i = 1)$ and $N_j = \sum_{i=1}^n I(t_{(j)} \le \tilde{T}_i < t_{(j+1)}, \delta_i = 0)$ as the number of failure time points at time $t_{(j)}$ and the number of censored time points in the interval $[t_{(j)}, t_{(j+1)})$, respectively. To estimate $F(t)$ nonparametrically, it assumes that $F(t)$ takes jumps only on uncensored failure times. Under the model assumption, the log-likelihood function can be written as

$$\begin{aligned}
&\log L(\theta, F(t)) \\
&= \sum_{i=1}^{D} \{M_i \log[S(t_{i-}) - S(t_{(i)})] + N_i \log(S(t_{(i)}))\} \\
&= -\theta \sum_{i=2}^{D} \sum_{k=1}^{i-1} M_i \cdot J_k + \sum_{i=1}^{D} M_i \log[1 - \exp(-\theta J_i)] - \theta \sum_{i=1}^{D} \sum_{k=1}^{i} N_i \cdot J_k, \qquad \text{(A-1)}
\end{aligned}$$

where $J_k = F(t_{(k)}) - F(t_{(k-1)})$ with restriction $\sum_{k=1}^{D} J_k = 1$. Tsodikov [1] proposed to use Lagrange multiplier method to obtain estimators for θ and $J_k, k = 1, ..., D$. Here, to obtain estimates, we consider to adopt the change of variables approach under the condition $\sum_{k=1}^{D} J_k = 1$.

Define $\theta_k = \theta \cdot J_k$ for $k = 1, ..., D$. The log-likelihood function (A-1) can be expressed as

$$\log L(\theta_1, ..., \theta_D) \tag{A-2}$$
$$= -\sum_{i=2}^{D}\sum_{k=1}^{i-1} M_i \cdot \theta_k + \sum_{i=1}^{D} M_i \log[1 - \exp(-\theta_i)] - \sum_{i=1}^{D}\sum_{k=1}^{i} N_i \cdot \theta_k.$$

Thus, to obtain estimates, one can sequentially solve the score function relating to $\theta_k, k = 1, ..., D$. Specifically, let $\hat{\theta}_D$ be the estimate by solving the score function

$$\frac{\partial \log L(\theta_1, ..., \theta_D)}{\partial \theta_D} = 0 \Leftrightarrow \frac{M_D \exp(-\theta_D)}{1 - \exp(-\theta_D)} - N_D = 0.$$

This implies that

$$\hat{\theta}_D = -\log \frac{N_D}{N_D + M_D}.$$

Also, let $\hat{\theta}_k, k = 1, ..., D-1$ be the estimate by solving the following score function

$$\frac{M_k \exp(-\theta_k)}{1 - \exp(-\theta_k)} - \sum_{\ell=k+1}^{D} M_\ell - \sum_{\ell=k}^{D} N_\ell = 0, k = 1, ..., D-1,$$

respectively. This indicates that

$$\hat{\theta}_k = -\log\left(\frac{\sum_{\ell=k+1}^{D} M_\ell + \sum_{\ell=k}^{D} N_\ell}{\sum_{\ell=k}^{D} M_\ell + \sum_{\ell=k}^{D} N_\ell}\right), k = 1, ..., D-1.$$

As we have $\theta_1 + ... + \theta_D = \theta \cdot (J_1 + ... + J_D) = \theta$, this implies that θ can then be estimated by $\hat{\theta}_{\text{NP}} = \sum_{k=1}^{D} \hat{\theta}_k$. Also, the jump size J_k can be estimated by $\hat{J}_k = \dfrac{\hat{\theta}_k}{\hat{\theta}_{\text{NP}}}$, $k = 1, ..., D$. Finally, the function $F(t)$ can then be estimated by

$$\hat{F}_{\text{NP}}(t) = \sum_{\{j: t_{(j)} \leq t\}} \hat{J}_j. \tag{A-3}$$

Web Appendix B

Figure 1 presents the box plots of the pseudo-observations based on simulated datasets with various sample size created by the estimators $\tilde{\pi}^i_{\text{KM}}$ and $\tilde{\pi}^i_{\text{NP}}$ from Equations (3.6) and (3.7) in the main manuscript, respectively. The data are generated from PHMC model consisting of the logistic regression model (2.2) and the Cox PH model (2.3) as stated in the Simulation section of the main manuscript. We set $\boldsymbol{\beta} = (1, 0.5)$ and $\boldsymbol{\alpha} = (2, -1)$ with covariates generated as described in the Simulation section of the main manuscript. The censoring rate is set to be 30%.

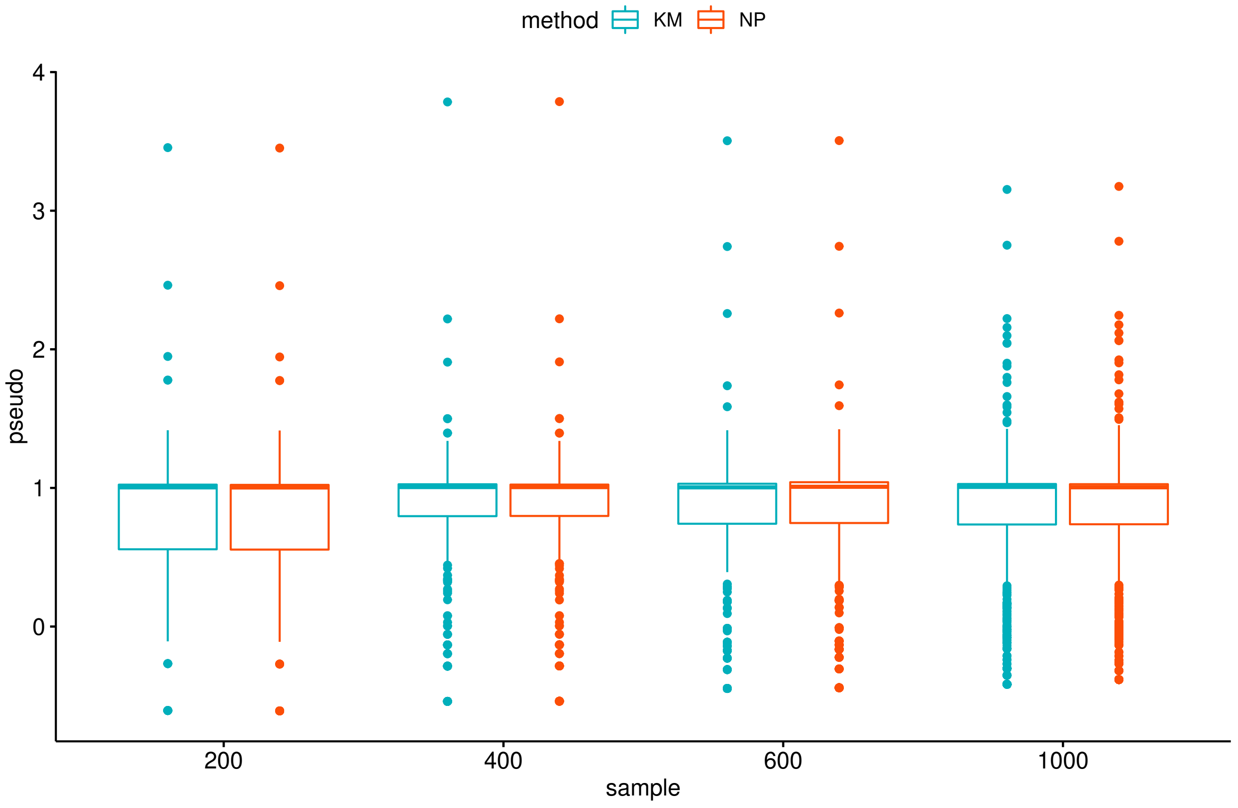

Figure 1: The pseudo-observations for $\hat{\pi}^i_{\mathrm{KM}}$ and $\hat{\pi}^i_{\mathrm{NP}}$ with various sample size.

Web Appendix C

In this section, we provide proofs of the asymptotic unbiased property for our proposed pseudo-observations given covariates. Let $(\mathbf{D}, ||\cdot||)$ and $(\mathbf{E}, ||\cdot||)$ be Banach spaces and $(\Omega, \mathcal{F}, P)$ be a probability space. Consider an open subset $W \subseteq \boldsymbol{D}$ and a functional $\phi : W \to \boldsymbol{E}$. Assume that $(\mathcal{X}, \mathcal{A})$ is a measure space. Define a map $\delta_{(\cdot)} : \mathcal{X} \longrightarrow \mathbf{D}$ and consider an i.i.d. sample $\boldsymbol{Q} = (Q_1, ..., Q_n)$ defined on $(\Omega, \mathcal{F}, P)$ with values in $\mathcal{X}$. We then define the sample average by $\mathbb{F}_n = \dfrac{1}{n}\sum_{i=1}^{n} \delta_{Q_i} \in \mathbf{D}$, and denote $\mathbb{F} \in \mathbf{D}$ as the limit of $\mathbb{F}_n$ if it does exist.

First, we impose the following regularity conditions and results obtained from Jacobsen and Martinussen [2] and Overgaard et al.[3].

(I.1) The Kaplan-Meier functional ψ and Nelson-Aalen functional ϕ are C^k for any $k \in \mathbb{N}$; that is, ψ and ϕ are k times continuously differentiable.

(I.2) There exists a constant d and $\lambda \in [\frac{1}{4}, \frac{1}{2})$ such that $||\delta_Q|| \leq d$ and $||\mathbb{F}_n - \mathbb{F}|| = o_p(n^{-\lambda})$.

(I.3) $E[\dot{\psi}(Q; t)] = 0$ and $E[\ddot{\psi}(Q_i, q; t)] = 0$ for all $q \in \mathcal{X}$, where $\dot{\psi}(\cdot; t)$ and $\ddot{\psi}(\cdot; t)$ are the first and second order bounded influence functions of the functional ψ evaluated at time t. Similarly, $E[\dot{\phi}(Q; t)] = 0$ and $E[\ddot{\phi}(Q_i, q; t)] = 0$ for all $q \in \mathcal{X}$, where $\dot{\phi}(\cdot; t)$ and $\ddot{\phi}(\cdot; t)$ are the first and second order bounded influence functions of the functional ϕ evaluated at time t.

(I.4) Covariates $\boldsymbol{Z}_i$ and $\mathbf{X}_i$ are bounded; i.e., there exists intergers M_1 and M_2 such that $\|\mathbf{Z}_i\| \leq M_1$ and $\|\mathbf{X}_i\| \leq M_2$ for $i = 1, ..., n$.

Note that condition (I.2) is originally from equation (3.5) in Overgaard et al.[3] for which it guarantees the remainder converges to 0 as n goes to infinity.

Second, based on (I.1)-(I.4) and the results from Jacobsen and Martinussen [2] and Overgaard et al.[3], as the sample size $n \to \infty$, we have

$$E[\hat{S}^{i}_{\text{KM}}(t)|\boldsymbol{Z}_i] = E[n \cdot \hat{S}_{\text{KM}}(t) - (n-1) \cdot \hat{S}^{-i}_{\text{KM}}(t)|\boldsymbol{Z}_i] \approx S(t|\boldsymbol{Z}_i), \tag{C-1}$$
$$E[\hat{\Lambda}^{i}_{\text{NA}}(t)|\boldsymbol{Z}_i] = E[n \cdot \hat{\Lambda}_{\text{NA}}(t) - (n-1) \cdot \hat{\Lambda}^{-i}_{\text{NA}}(t)|\boldsymbol{Z}_i] \approx \Lambda(t|\boldsymbol{Z}_i), \tag{C-2}$$

where $\hat{S}_{\text{KM}}(t)$ is the Kaplan-Meier (KM) estimator for survival function $S(t)$, $\hat{\Lambda}_{\text{NA}}(t)$ is the Nelson-Aalen (NA) estimator for cumulative hazard function $\Lambda(t)$, $\hat{S}^{-i}_{\text{KM}}(t)$ and $\hat{\Lambda}^{-i}_{\text{NA}}(t)$ are the KM and NA estimators based on the remaining $n-1$ subjects, leaving subject i out from the sample, and $S(t|\boldsymbol{Z}_i)$ and $\Lambda(t|\boldsymbol{Z}_i)$ are survival and cumulative hazard functions given covariate $\boldsymbol{Z}_i$, respectively. Moreover, it is known that

$$\hat{S}_{\text{KM}}(t) \approx \exp(-\hat{\Lambda}_{\text{NA}}(t)) \tag{C-3}$$

as the sample size n goes to infinity.

Finally, the asymptotic unbiased property for our proposed pseudo-observations are shown in the following subsections.

C.1 The asymptotic unbiased for the pseudo-observations $\hat{\pi}^i_{\mathbf{KM}} = n \cdot \hat{\pi}_{\mathbf{KM}} - (n-1) \cdot \hat{\pi}^{-i}_{\mathbf{KM}}$

Proof. Under the mixture cure model assumption with covariates only have effects on the uncure rate, we have $S(\infty|\boldsymbol{X}_i) = 1 - \pi(\boldsymbol{X}_i)$. Based on Equation (C-1), $\hat{\pi}_{\text{KM}} = 1 - \hat{S}_{\text{KM}}(t_{\max})$ and $\hat{\pi}^{-i}_{\text{KM}} = 1 - \hat{S}^{-i}_{\text{KM}}(t_{\max})$, we have

$$\begin{aligned}
&E[\hat{\pi}^i_{\text{KM}}|\boldsymbol{X}_i] \\
&= E[n \cdot (1 - \hat{S}_{\text{KM}}(t_{\max})) - (n-1) \cdot (1 - \hat{S}^{-i}_{\text{KM}}(t_{\max}))|\boldsymbol{X}_i] \\
&= 1 - E[n \cdot \hat{S}_{\text{KM}}(\infty) - (n-1) \cdot \hat{S}^{-i}_{\text{KM}}(\infty)|\boldsymbol{X}_i] \\
&\approx 1 - S(\infty|\boldsymbol{X}_i) \\
&= \pi(\boldsymbol{X}_i) \qquad \text{(C-4)}
\end{aligned}$$

as $n \to \infty$. This completes the proof. □

C.2 The asymptotic unbiased for the pseudo-observations $\hat{\pi}^i_{\mathbf{NP}} = n \cdot \hat{\pi}_{\mathbf{NP}} - (n-1) \cdot \hat{\pi}^{-i}_{\mathbf{NP}}$

Proof. For $j = 1, ..., D$, recall that $M_j = \sum_{i=1}^n I(\tilde{T}_i = t_{(j)}, \delta_i = 1)$ and $N_j = \sum_{i=1}^n I(t_{(j)} \leq \tilde{T}_i < t_{(j+1)}, \delta_i = 0)$ are the number of failure times at time $t_{(j)}$ and the number of censored times in the interval $[t_{(j)}, t_{(j+1)})$, respectively. Based on Section 3.1 in the main text, we have θ can be estimated by $\hat{\theta}_{\text{NP}} = \sum_{k=1}^D \hat{\theta}_k$, where $\hat{\theta}_k = -\log\{(\sum_{\ell=k+1}^D M_\ell + \sum_{\ell=k}^D N_\ell)/(\sum_{\ell=k}^D M_\ell + \sum_{\ell=k}^D N_\ell)\}$, $k = 1, \ldots, D-1$, and $\hat{\theta}_D = -\log(N_D/(N_D + M_D))$. Therefore,

$$\begin{aligned}
&\hat{\pi}_{\text{NP}} = 1 - \exp(-\hat{\theta}_{\text{NP}}) \\
&= 1 - \exp(-\sum_{k=1}^D \hat{\theta}_k) \\
&= 1 - e^{-\hat{\theta}_1} \cdot e^{-\hat{\theta}_2} \cdot \cdots \cdot e^{-\hat{\theta}_D} \\
&= 1 - \left[\frac{\sum_{\ell=2}^D M_\ell + \sum_{\ell=1}^D N_\ell}{\sum_{\ell=1}^D M_\ell + \sum_{\ell=1}^D N_\ell} \cdot \frac{\sum_{\ell=3}^D M_\ell + \sum_{\ell=2}^D N_\ell}{\sum_{\ell=2}^D M_\ell + \sum_{\ell=2}^D N_\ell} \cdot \cdots \cdot \frac{\sum_{\ell=D}^D M_\ell + \sum_{\ell=D-1}^D N_\ell}{\sum_{\ell=D-1}^D M_\ell + \sum_{\ell=D-1}^D N_\ell} \cdot \frac{N_D}{N_D + M_D}\right] \\
&= 1 - \left[\frac{(\sum_{\ell=1}^D M_\ell + \sum_{\ell=1}^D N_\ell) - M_1}{\sum_{\ell=1}^D M_\ell + \sum_{\ell=1}^D N_\ell} \cdot \cdots \cdot \frac{(\sum_{\ell=D-1}^D M_\ell + \sum_{\ell=D-1}^D N_\ell) - M_{D-1}}{\sum_{\ell=D-1}^D M_\ell + \sum_{\ell=D-1}^D N_\ell} \cdot \frac{(N_D + M_D) - M_D}{N_D + M_D}\right] \\
&= 1 - \prod_{\{j: t_{(j)} \leq t_{\max}\}} \frac{n_j - M_j}{n_j} \\
&= 1 - \hat{S}_{\text{KM}}(t_{\max}) \\
&= 1 - \hat{S}_{\text{KM}}(\infty),
\end{aligned}$$

where $n_j = \sum_{\ell=j}^D M_\ell + \sum_{\ell=j}^D N_\ell$ is the number of subjects at risk at time $t_{(j)}$. Thus, based on the results from (C-4), we have

$$E[\hat{\pi}_{\text{NP}}|\boldsymbol{X}_i] \approx 1 - S(\infty|\boldsymbol{X}_i) = \pi(\boldsymbol{X}_i) \qquad \text{(C-5)}$$

as $n \to \infty$. This completes the proof. □

C.3 The asymptotic unbiased for the pseudo-observations $\hat{S}_u^i(t) = n \cdot \hat{S}_{u,\mathbf{KM}}(t) - (n-1) \cdot \hat{S}_{u,\mathbf{KM}}^{-i}(t)$

Proof. Under the mixture cure model assumption and covariates only have effects on the conditional survival function $S_u(t)$, we aim to show that $E[\hat{S}_u^i(t)|\boldsymbol{Z}_i] \approx S_u(t|\boldsymbol{Z}_i) = \dfrac{S(t|\boldsymbol{Z}_i) - (1-\pi)}{\pi}$ as $n \to \infty$, where $\pi = P(Y=1) = 1 - S(\infty)$. Write

$$
\begin{aligned}
& n \cdot \hat{S}_{u,\mathrm{KM}}(t) - (n-1) \cdot \hat{S}_{u,\mathrm{KM}}^{-i}(t) \\
&= n \cdot \frac{\hat{S}_{\mathrm{KM}}(t) - \hat{S}_{\mathrm{KM}}(t_{\max})}{1 - \hat{S}_{\mathrm{KM}}(t_{\max})} - (n-1) \cdot \frac{\hat{S}_{\mathrm{KM}}^{-i}(t) - \hat{S}_{\mathrm{KM}}^{-i}(t_{\max})}{1 - \hat{S}_{\mathrm{KM}}^{-i}(t_{\max})} \\
&= n \cdot \frac{\hat{S}_{\mathrm{KM}}(t) - \hat{S}_{\mathrm{KM}}(\infty)}{1 - \hat{S}_{\mathrm{KM}}(\infty)} - \frac{(n-1) \cdot [\hat{S}_{\mathrm{KM}}^{-i}(t) - \hat{S}_{\mathrm{KM}}^{-i}(\infty)]}{1 - \hat{S}_{\mathrm{KM}}(\infty)} + \frac{(n-1) \cdot [\hat{S}_{\mathrm{KM}}^{-i}(t) - \hat{S}_{\mathrm{KM}}^{-i}(\infty)]}{1 - \hat{S}_{\mathrm{KM}}(\infty)} \\
&- (n-1) \cdot \frac{\hat{S}_{\mathrm{KM}}^{-i}(t) - \hat{S}_{\mathrm{KM}}^{-i}(\infty)}{1 - \hat{S}_{\mathrm{KM}}^{-i}(\infty)} \\
&= \frac{1}{1 - \hat{S}_{\mathrm{KM}}(\infty)} \cdot \Big\{ \big(n \cdot \hat{S}_{\mathrm{KM}}(t) - (n-1)\hat{S}_{\mathrm{KM}}^{-i}(t)\big) - \big(n \cdot \hat{S}_{\mathrm{KM}}(\infty) - (n-1) \cdot \hat{S}_{\mathrm{KM}}^{-i}(\infty)\big) \Big\} \\
&+ (n-1) \cdot \Big(\hat{S}_{\mathrm{KM}}^{-i}(t) - \hat{S}_{\mathrm{KM}}^{-i}(\infty) \Big) \cdot \Big[\frac{\hat{S}_{\mathrm{KM}}(\infty) - \hat{S}_{\mathrm{KM}}^{-i}(\infty)}{(1 - \hat{S}_{\mathrm{KM}}(\infty)) \cdot (1 - \hat{S}_{\mathrm{KM}}^{-i}(\infty))} \Big] \\
&= \frac{1}{1 - S(\infty)} \cdot \Big\{ \big(n \cdot \hat{S}_{\mathrm{KM}}(t) - (n-1)\hat{S}_{\mathrm{KM}}^{-i}(t)\big) - \big(n \cdot \hat{S}_{\mathrm{KM}}(\infty) - (n-1) \cdot \hat{S}_{\mathrm{KM}}^{-i}(\infty)\big) \Big\} \quad \text{(C-6)} \\
&+ \Big(\frac{1}{1 - \hat{S}_{\mathrm{KM}}(t)} - \frac{1}{1 - S(\infty)} \Big) \Big\{ \big(n \cdot \hat{S}_{\mathrm{KM}}(t) - (n-1)\hat{S}_{\mathrm{KM}}^{-i}(t)\big) - \big(n \cdot \hat{S}_{\mathrm{KM}}(\infty) - (n-1) \cdot \hat{S}_{\mathrm{KM}}^{-i}(\infty)\big) \Big\} \\
& \quad \text{(C-7)} \\
&+ (n-1) \cdot \Big(\hat{S}_{\mathrm{KM}}^{-i}(t) - \hat{S}_{\mathrm{KM}}^{-i}(\infty) \Big) \cdot \Big[\frac{\hat{S}_{\mathrm{KM}}(\infty) - \hat{S}_{\mathrm{KM}}^{-i}(\infty)}{(1 - \hat{S}_{\mathrm{KM}}(\infty)) \cdot (1 - \hat{S}_{\mathrm{KM}}^{-i}(\infty))} \Big]. \quad \text{(C-8)}
\end{aligned}
$$

In Equation (C-7), when $n \to \infty$, the term

$$
\begin{aligned}
\frac{1}{1 - \hat{S}_{\mathrm{KM}}(t)} - \frac{1}{1 - S(\infty)} &= \frac{\hat{S}_{\mathrm{KM}}(\infty) - S(\infty)}{1 - S(\infty) - \hat{S}_{\mathrm{KM}}(\infty) + \hat{S}_{\mathrm{KM}}(\infty)S(\infty)} \\
&\leq \frac{\hat{S}_{\mathrm{KM}}(\infty) - S(\infty)}{1 - S(\infty) - \hat{S}_{\mathrm{KM}}(\infty)} \\
&\to 0 \qquad \text{(C-9)}
\end{aligned}
$$

as the KM estimator $\hat{S}_{\mathrm{KM}}(t)$ is a consistent estimator of $S(t)$. Moreover, based on the von Mises expansion and the results from Jacobsen and Martinussen [2], we have

$$
\begin{aligned}
& \big(n \cdot \hat{S}_{\mathrm{KM}}(t) - (n-1)\hat{S}_{\mathrm{KM}}^{-i}(t)\big) - \big(n \cdot \hat{S}_{\mathrm{KM}}(\infty) - (n-1) \cdot \hat{S}_{\mathrm{KM}}^{-i}(\infty)\big) \\
& \approx S(t) - S(\infty) + \dot{\psi}(\tilde{T}_i; t) - \dot{\psi}(\tilde{T}_i; \infty) \qquad \text{(C-10)}
\end{aligned}
$$

which is bounded due to the fact that the survival function $S(t)$ and the first-order influence function $\dot{\psi}(\tilde{T}_i; t) = -S(t) \cdot \int_0^t \frac{1}{H(s)} dM_i(s)$ are bounded (Condition (I.3)), where $M_i(t) = I(\tilde{T}_i \leq t, \delta_i = 1) - \int_0^t I(\tilde{T}_i \geq s) d\Lambda(s)$, $H(s) = S(s) \cdot G(s)$ and $G(\cdot)$ is the survival function

for censoring time. Thus, Equation (C-7) goes to 0 as $n \to \infty$. On the other hand, for Equation (C-8), we have

$$
\begin{aligned}
&(n-1)\cdot\Big(\hat{S}_{\text{KM}}^{-i}(t)-\hat{S}_{\text{KM}}^{-i}(\infty)\Big)\cdot\Big[\frac{\hat{S}_{\text{KM}}(\infty)-\hat{S}_{\text{KM}}^{-i}(\infty)}{(1-\hat{S}_{\text{KM}}(\infty))\cdot(1-\hat{S}_{\text{KM}}^{-i}(\infty))}\Big]\\
&\le (n-1)\cdot(1-\hat{S}_{\text{KM}}^{-i}(\infty))\cdot\Big[\frac{\hat{S}_{\text{KM}}(\infty)-\hat{S}_{\text{KM}}^{-i}(\infty)}{(1-\hat{S}_{\text{KM}}(\infty))\cdot(1-\hat{S}_{\text{KM}}^{-i}(\infty))}\Big]\\
&=(n-1)\cdot\frac{\hat{S}_{\text{KM}}(\infty)-\hat{S}_{\text{KM}}^{-i}(\infty)}{(1-\hat{S}_{\text{KM}}(\infty))}. \qquad \text{(C-11)}
\end{aligned}
$$

In addition, based on the von Mises expansion, Condition (I.3) and the results from Jacobsen and Martinussen [2] and Overgaard et al. [3], we have

$$
\begin{aligned}
&(n-1)\cdot[\hat{S}_{\text{KM}}(\infty)-\hat{S}_{\text{KM}}^{-i}(\infty)]\\
&=(n-1)\cdot\Big[\hat{S}_{\text{KM}}(\infty)-S(\infty)\Big]-(n-1)\cdot\Big[\hat{S}_{\text{KM}}^{-i}(\infty)-S(\infty)\Big]\\
&\approx n\cdot\Big[\frac{1}{n}\sum_{i=1}^{n}\dot{\psi}(\tilde{T}_i;\infty)+\frac{1}{2n^2}\sum_{i=1}^{n}\sum_{j=1}^{n}\ddot{\psi}(\tilde{T}_i,\tilde{T}_j;\infty)\Big]\\
&-(n-1)\cdot\Big[\frac{1}{n-1}\sum_{\ell\neq i}^{n}\dot{\psi}(\tilde{T}_\ell;\infty)+\frac{1}{2(n-1)^2}\sum_{\ell\neq i}^{n}\sum_{k\neq i}^{n}\ddot{\psi}(\tilde{T}_\ell,\tilde{T}_k;\infty)\Big]\\
&=\sum_{i=1}^{n}\dot{\psi}(\tilde{T}_i;\infty)+\frac{1}{2n}\sum_{i=1}^{n}\sum_{j=1}^{n}\ddot{\psi}(\tilde{T}_i,\tilde{T}_j;\infty)-\sum_{\ell\neq i}^{n}\dot{\psi}(\tilde{T}_\ell;\infty)-\frac{1}{2(n-1)}\sum_{\ell\neq i}^{n}\sum_{k\neq i}^{n}\ddot{\psi}(\tilde{T}_\ell,\tilde{T}_k;\infty)\\
&=\dot{\psi}(\tilde{T}_i;\infty)+\frac{1}{n-1}\sum_{\ell\neq k}\ddot{\psi}(\tilde{T}_\ell,\tilde{T}_k;\infty)\\
&=o_p(n) \qquad \text{(C-12)}
\end{aligned}
$$

because $|\dot{\psi}(\tilde{T}_i;\infty)|$ is bounded and $|(n-1)^{-1}\sum_{\ell\neq k}\ddot{\psi}(\tilde{T}_\ell,\tilde{T}_k;\infty)|$ converge to 0 for $n\to\infty$, where $\ddot{\psi}(\tilde{T}_i,\tilde{T}_j;\infty)$ is the second-order influence function of the Kaplan-Meier functional evaluated at time ∞ (See Section 4.1 of Overgaard et al.[3]). Thus, based on above results along with Equation (C-1), we obtain

$$
\begin{aligned}
&E[n\cdot\hat{S}_{u,\text{KM}}(t)-(n-1)\cdot\hat{S}_{u,\text{KM}}^{-i}(t)|\boldsymbol{Z}_i]\\
&\approx\frac{1}{1-S(\infty)}\cdot E\Big[\big(n\cdot\hat{S}_{\text{KM}}(t)-(n-1)\hat{S}_{\text{KM}}^{-i}(t)\big)-\big(n\cdot\hat{S}_{\text{KM}}(\infty)-(n-1)\cdot\hat{S}_{\text{KM}}^{-i}(\infty)\big)\Big|\boldsymbol{Z}_i\Big]\\
&=\frac{1}{\pi}\cdot[S(t|\boldsymbol{Z}_i)-S(\infty)]\\
&=\frac{S(t|\boldsymbol{Z}_i)-(1-\pi)}{\pi}
\end{aligned}
$$

as $n\to\infty$. This completes the proof. □

C.4 The asymptotic unbiased for the pseudo-observations $\hat{\theta}^i_{\mathbf{KM}} = n\cdot\hat{\theta}_{\mathbf{KM}} - (n-1)\cdot\hat{\theta}^{-i}_{\mathbf{KM}}$

Proof. Recall that under the BCH model (2.4) defined in the main manuscript, we have $S(t)=\exp(-\theta F(t))$, where $F(t)=\Lambda(t)/\theta$ is a proper cumulative distribution function of

a nonnegative random variable with $\Lambda(\infty) = \theta > 0$, $F(0) = 0$ and $F(\infty) = \Lambda(\infty)/\theta = 1$. Denote $\Lambda(t|\boldsymbol{X}_i)$ as the cumulative hazard function given covariate $\boldsymbol{X}_i$. Based on Equations (C-2), (C-3) and $\hat{\theta}_{\text{KM}} = -\log \hat{S}_{\text{KM}}(t_{\max}) = -\log \hat{S}_{\text{KM}}(\infty)$, we have

$$\begin{aligned}
&E[\hat{\theta}^i_{\text{KM}}|\boldsymbol{X}_i] \\
&= E[n \cdot \hat{\theta}_{\text{KM}} - (n-1) \cdot \hat{\theta}^{-i}_{\text{KM}}|\boldsymbol{X}_i] \\
&= E[n \cdot -\log \hat{S}_{\text{KM}}(\infty) + (n-1) \cdot \log \hat{S}^{-i}_{\text{KM}}(\infty)|\boldsymbol{X}_i] \\
&\approx E[n \cdot \hat{\Lambda}_{\text{NA}}(\infty) - (n-1) \cdot \hat{\Lambda}^{-i}_{\text{NA}}(\infty)|\boldsymbol{X}_i] \\
&= \Lambda(\infty|\boldsymbol{X}_i) \\
&= \theta(\boldsymbol{X}_i)
\end{aligned}$$

as $n \to \infty$. This completes the proof. □

C.5 The asymptotic unbiased for the pseudo-observations $\hat{\theta}^i_{\mathbf{NP}} = n \cdot \hat{\theta}_{\mathbf{NP}} - (n-1) \cdot \hat{\theta}^{-i}_{\mathbf{NP}}$

Proof. From Section C.2, we obtain that $1 - \exp(-\hat{\theta}_{\text{NP}}) = 1 - \hat{S}_{\text{KM}}(t_{\max}) = 1 - \hat{S}_{\text{KM}}(\infty)$. Moreover, with the results from Equation (C-3), we obtain $\hat{\theta}_{\text{NP}} \approx \hat{\Lambda}_{\text{NA}}(t_{\max}) = \hat{\Lambda}_{\text{NA}}(\infty)$. Thus, based on Equation (C-2), we have

$$E[\hat{\theta}^i_{\text{NP}}|\boldsymbol{X}_i] \approx E[n \cdot \hat{\Lambda}_{\text{NA}}(\infty) - (n-1) \cdot \hat{\Lambda}^{-i}_{\text{NA}}(\infty)|\boldsymbol{X}_i] = \Lambda(\infty|\boldsymbol{X}_i) = \theta(\boldsymbol{X}_i). \tag{C-13}$$

This completes the proof. □

C.6 The asymptotic unbiased for the pseudo-observations $\hat{F}^i_{\mathbf{KM}}(t) = n \cdot \hat{F}_{\mathbf{KM}}(t) - (n-1) \cdot \hat{F}^{-i}_{\mathbf{KM}}(t)$

Proof. To prove $E[n \cdot (1 - \hat{F}_{\text{KM}}(t)) - (n-1) \cdot (1 - \hat{F}^{-i}_{\text{KM}})(t)|\boldsymbol{Z}_i] \approx 1 - \dfrac{\Lambda(t|\boldsymbol{Z}_i)}{\theta}$ as $n \to \infty$, it is equivalent to show that $E[n \cdot \hat{F}_{\text{KM}}(t) - (n-1) \cdot \hat{F}^{-i}_{\text{KM}}(t)|\boldsymbol{Z}_i] \approx \dfrac{\Lambda(t|\boldsymbol{Z}_i)}{\theta}$ as $n \to \infty$. Based on Equation (C-3), we write

$$\begin{aligned}
&n \cdot \hat{F}_{\text{KM}}(t) - (n-1) \cdot \hat{F}^{-i}_{\text{KM}}(t) \\
&= n \cdot \frac{\log \hat{S}_{\text{KM}}(t)}{\log \hat{S}_{\text{KM}}(t_{\max})} - (n-1) \cdot \frac{\log \hat{S}^{-i}_{\text{KM}}(t)}{\log \hat{S}^{-i}_{\text{KM}}(t_{\max})} \\
&= n \cdot \frac{\log \hat{S}_{\text{KM}}(t)}{\log \hat{S}_{\text{KM}}(\infty)} - (n-1) \cdot \frac{\log \hat{S}^{-i}_{\text{KM}}(t)}{\log \hat{S}^{-i}_{\text{KM}}(\infty)} \\
&\approx n \cdot \frac{\hat{\Lambda}_{\text{NA}}(t)}{\hat{\Lambda}_{\text{NA}}(\infty)} - (n-1) \cdot \frac{\hat{\Lambda}^{-i}_{\text{NA}}(t)}{\hat{\Lambda}^{-i}_{\text{NA}}(\infty)} \qquad \text{(By (C-3))} \\
&= n \cdot \frac{\hat{\Lambda}_{\text{NA}}(t)}{\hat{\Lambda}_{\text{NA}}(\infty)} - (n-1) \cdot \frac{\hat{\Lambda}^{-i}_{\text{NA}}(t)}{\hat{\Lambda}_{\text{NA}}(\infty)} + (n-1) \cdot \frac{\hat{\Lambda}^{-i}_{\text{NA}}(t)}{\hat{\Lambda}_{\text{NA}}(\infty)} - (n-1) \cdot \frac{\hat{\Lambda}^{-i}_{\text{NA}}(t)}{\hat{\Lambda}^{-i}_{\text{NA}}(\infty)} \\
&= \frac{1}{\hat{\Lambda}_{\text{NA}}(\infty)} \left[n \cdot \hat{\Lambda}_{\text{NA}}(t) - (n-1) \cdot \hat{\Lambda}^{-i}_{\text{NA}}(t) \right] + (n-1) \cdot \hat{\Lambda}^{-i}_{\text{NA}}(t) \cdot \left[\frac{1}{\hat{\Lambda}_{\text{NA}}(\infty)} - \frac{1}{\hat{\Lambda}^{-i}_{\text{NA}}(\infty)} \right].
\end{aligned} \tag{C-14}$$

Write the first term of Equation (C-14) as

$$\frac{1}{\hat{\Lambda}_{\text{NA}}(\infty)}\Big[n\cdot\hat{\Lambda}_{\text{NA}}(t)-(n-1)\cdot\hat{\Lambda}_{\text{NA}}^{-i}(t)\Big]$$

$$=\frac{1}{\Lambda(\infty)}\cdot\Big[n\cdot\hat{\Lambda}_{\text{NA}}(t)-(n-1)\cdot\hat{\Lambda}_{\text{NA}}^{-i}(t)\Big] \tag{C-15}$$

$$+\Big[\frac{1}{\hat{\Lambda}_{\text{NA}}(\infty)}-\frac{1}{\Lambda(\infty)}\Big]\cdot\Big[n\cdot\hat{\Lambda}_{\text{NA}}(t)-(n-1)\cdot\hat{\Lambda}_{\text{NA}}^{-i}(t)\Big]. \tag{C-16}$$

For Equation (C-16), based on the von Mises expansion, Conditions (I.2) and (I.3) and the results from Overgaard et al.[3], we have

$$\Big[\frac{1}{\hat{\Lambda}_{\text{NA}}(\infty)}-\frac{1}{\Lambda(\infty)}\Big]\cdot\Big[n\cdot\hat{\Lambda}_{\text{NA}}(t)-(n-1)\cdot\hat{\Lambda}_{\text{NA}}^{-i}(t)\Big]$$

$$\approx\frac{\Lambda(\infty)-\hat{\Lambda}_{\text{NA}}(\infty)}{\hat{\Lambda}_{\text{NA}}(\infty)\Lambda(\infty)}\cdot\Big[\Lambda(t)+\dot{\phi}(\tilde{T}_i;t)+\frac{1}{n-1}\sum_{\ell\neq k}\ddot{\phi}(\tilde{T}_\ell,\tilde{T}_k;t)\Big]$$

$$\leq\theta^{-2}\cdot[\Lambda(\infty)-\hat{\Lambda}_{\text{NA}}(\infty)]\cdot\Big[\Lambda(t)+K\cdot(d+\Lambda(t))+\frac{1}{n-1}\sum_{\ell\neq k}\ddot{\phi}(\tilde{T}_\ell,\tilde{T}_k;t)\Big]$$

$$\longrightarrow 0\quad\text{as}\quad n\to\infty, \tag{C-17}$$

where $K>0$ is a constant, $\dot{\phi}(\tilde{T}_i;t)$ and $\ddot{\phi}(\tilde{T}_\ell,\tilde{T}_k;t)$ are the first and second-order bounded influence functions of the Nelson-Aalen functional as presented in the Example 2.2 of Overgaard et al. [3]. Note that $\Lambda(\infty)-\hat{\Lambda}_{\text{NA}}(\infty)$ and $(n-1)^{-1}\sum_{\ell\neq k}\ddot{\phi}(\tilde{T}_\ell,\tilde{T}_k;t)$ converge to 0 as $n\to\infty$.

On the other hand, based on the same idea from (C-12), we can show that the order of the second term of Equation (C-14) is $o_p(n)$. Thus, based on (C-2), (C-14), (C-15), (C-16) and (C-17), we obtain

$$E[n\cdot\hat{F}_{\text{KM}}(t)-(n-1)\cdot\hat{F}_{\text{KM}}^{-i}(t)|\boldsymbol{Z}_i]$$

$$\approx\frac{1}{\Lambda(\infty)}\cdot E[n\cdot\hat{\Lambda}_{\text{NA}}(t)-(n-1)\cdot\hat{\Lambda}_{\text{NA}}^{-i}(t)|\boldsymbol{Z}_i]$$

$$=\frac{\Lambda(t|\boldsymbol{Z}_i)}{\theta}$$

as $n\to\infty$. This completes the proof.

□

C.7 The asymptotic unbiased for the pseudo-observations $\hat{F}_{\mathbf{NP}}^i(t)=n\cdot\hat{F}_{\mathbf{NP}}(t)-(n-1)\cdot\hat{F}_{\mathbf{NP}}^{-i}(t)$

Proof. Based on the results in Section C.5, we have $\theta_{\text{NP}}\approx\hat{\Lambda}_{\text{NA}}(\infty)$, which leads to $\hat{F}_{\text{NP}}(t)=\dfrac{\sum_{\{j:t_{(j)}\leq t\}}\hat{\theta}_j}{\hat{\theta}_{\text{NP}}}\approx\dfrac{\hat{\Lambda}_{\text{NA}}(t)}{\hat{\Lambda}_{\text{NA}}(\infty)}$. Therefore, the asymptotic unbiased of the pseudo-observations $\hat{F}_{\text{NP}}^i(t)$ given covariates $\boldsymbol{Z}_i$ directly follows the results from Section C.6. This completes the proof. □

Web Appendix D

In this Web Appendix, we provide the data generation algorithm for the PHPH model mentioned in the main manuscript. We aim to generate survival time T_0 from the improper survival function $S(t) = \exp\{-\theta(\boldsymbol{X})(1 - \bar{F}(t)^{\eta(\boldsymbol{Z})})\}$ with $\theta(\boldsymbol{X}) = \exp(\gamma_0 + \boldsymbol{\gamma}^\top \boldsymbol{X})$, $\eta(\boldsymbol{Z}) = \exp(\boldsymbol{\phi}^\top \boldsymbol{Z})$ and an exponential survival function $\bar{F}(t) = \exp(-\kappa \cdot t)$. The main idea is to utilize the connection between the mixture cure (MC) model and the PHPH model. Under the MC model, we have $S(t) = (1-\pi)+\pi \cdot S_u(t)$, where $S_u(t)$ is a proper conditional survival function of a failure time variable T^*. Thus, $S_u(t)$ can be expressed as

$$\begin{aligned} S_u(t) &= \frac{\exp(-\theta(\boldsymbol{X})(1 - \bar{F}(t)^{\eta(\boldsymbol{Z})})) - (1-\pi)}{\pi} \\ &= \frac{\exp(-\theta(\boldsymbol{X})(1 - \bar{F}(t)^{\eta(\boldsymbol{Z})})) - \exp(-\theta(\boldsymbol{X}))}{1 - \exp(-\theta(\boldsymbol{X}))}. \end{aligned} \tag{D-1}$$

Based on (D-1) and $S_u(T^*)$ follows a Uniform distribution over (0,1), we have

$$\log \bar{F}(t) = \frac{1}{\eta(\boldsymbol{Z})} \log \left\{ 1 + \frac{1}{\theta(\boldsymbol{X})} \log \Big[U \cdot (1 - \exp(-\theta(\boldsymbol{X}))) + \exp(-\theta(\boldsymbol{X})) \Big] \right\},$$

where U follows a Uniform distribution over (0,1). Thus, the data generation algorithm can then be summarized as follows.

Step 1 : Generate U from a Uniform distribution over (0,1).

Step 2 : Let $T^{**} = \frac{-1}{\kappa \cdot \eta(\boldsymbol{Z})} \log \left\{ 1 + \frac{1}{\theta(\boldsymbol{X})} \cdot \log \left[U \cdot (1 - \exp(-\boldsymbol{\theta}(\boldsymbol{X}))) + \exp(-\theta(\boldsymbol{X})) \right] \right\}$.

Step 3 : Generate V from a Uniform distribution over (0,1).

Step 4 : If $V > \exp(-\theta(\boldsymbol{X}))$, we set $T_0 = T^{**}$. If $V < \exp(-\theta(\boldsymbol{X}))$, we set T_0 to be a large number.

Web Appendix E

In this Appendix, we provide the extra tables mentioned in the main manuscript.

Table 1: Simulation summaries under the PHMC model based on 500 replicates.

		Incidence						Latency			
n		$\hat{\alpha}^{\text{PO}}_{0,\text{NP}}$	$\hat{\alpha}^{\text{PO}}_{1,\text{NP}}$	$\hat{\alpha}^{\text{PO}}_{0,\text{KM}}$	$\hat{\alpha}^{\text{PO}}_{1,\text{KM}}$	$\hat{\alpha}^{\text{EM}}_{0}$	$\hat{\alpha}^{\text{EM}}_{1}$	$\hat{\beta}^{\text{PO}}_{1}$	$\hat{\beta}^{\text{PO}}_{2}$	$\hat{\beta}^{\text{EM}}_{1}$	$\hat{\beta}^{\text{EM}}_{2}$
		10% cure rate, 20% censoring rate									
200	Bias	0.018	0.012	0.019	0.012	0.031	0.005	0.022	-0.003	0.019	0.005
	ESE	0.602	0.739	0.604	0.737	0.520	0.611	0.226	0.362	0.202	0.303
	SEE	0.699	0.857	0.700	0.849	0.663	0.728	0.218	0.344	0.191	0.308
	CR	0.924	0.974	0.924	0.972	0.926	0.966	0.946	0.944	0.942	0.950
400	Bias	0.022	-0.023	0.026	-0.025	0.032	-0.013	0.016	-0.000	0.012	-0.000
	ESE	0.449	0.516	0.449	0.517	0.385	0.441	0.148	0.256	0.128	0.220
	SEE	0.475	0.549	0.476	0.551	0.397	0.458	0.147	0.242	0.129	0.211
	CR	0.946	0.972	0.950	0.974	0.954	0.974	0.952	0.930	0.932	0.940
600	Bias	0.056	-0.034	0.050	-0.031	0.055	-0.036	0.006	-0.008	0.006	-0.014
	ESE	0.392	0.458	0.383	0.451	0.354	0.406	0.113	0.193	0.102	0.166
	SEE	0.404	0.468	0.400	0.465	0.429	0.503	0.121	0.199	0.106	0.171
	CR	0.960	0.972	0.964	0.968	0.942	0.970	0.964	0.956	0.950	0.948
1000	Bias	-0.012	-0.001	0.025	-0.024	0.023	-0.016	0.001	-0.004	0.002	-0.004
	ESE	0.312	0.347	0.329	0.369	0.261	0.301	0.086	0.157	0.077	0.138
	SEE	0.308	0.339	0.315	0.361	0.244	0.300	0.093	0.154	0.081	0.132
	CR	0.940	0.950	0.946	0.958	0.934	0.954	0.972	0.948	0.958	0.936

Bias: bias of parameter estimator; ESE: the empirical standard error; SEE: average of the standard error estimator; CR: coverage rate of the 95% confidence interval; $(\hat{\alpha}^{\text{EM}}_{0}, \hat{\alpha}^{\text{EM}}_{1})$ and $(\hat{\beta}^{\text{EM}}_{1}, \hat{\beta}^{\text{EM}}_{2})$ are EM-algorithm based estimators with standard errors are estimated based on $B = 100$ bootstrap samples [4] which can be implemented via the R package *smcure* [5].

Table 2: Comparison of computing time under the mixture cure model between Peng and Dear [4] and our proposed estimators using pseudo-observations based on 500 replicates.

		Proposed Method				
			NP Method		KM Method	
n	smcure	Latency	Incidence	Total	Incidence	Total
200	7.47	0.20	0.37	0.57	0.17	0.37
400	11.58	0.46	1.46	1.92	0.44	0.90
600	16.16	0.76	3.29	4.05	0.78	1.54
1000	30.03	1.64	9.82	11.46	1.75	3.39

smcure: EM-algorithm estimators with standard errors are estimated based on $B = 100$ bootstrap samples [4] which can be implemented via the R package *smcure* [5].

Table 3: Simulation summaries for the proposed estimators of the *long-term* effect under PHPH model.

(n,Cure,Cen.)		PO				MLE	
		$\hat{\gamma}_{0,\mathrm{NP}}^{\mathrm{PO}}$	$\hat{\gamma}_{1,\mathrm{NP}}^{\mathrm{PO}}$	$\hat{\gamma}_{0,\mathrm{KM}}^{\mathrm{PO}}$	$\hat{\gamma}_{1,\mathrm{KM}}^{\mathrm{PO}}$	$\hat{\gamma}_{0}^{\mathrm{MLE}}$	$\hat{\gamma}_{1}^{\mathrm{MLE}}$
(200,20%,30%)	Bias	-0.030	-0.013	-0.029	-0.014	-0.002	-0.011
	ESE	0.159	0.244	0.159	0.244	0.126	0.195
	SEE	0.154	0.234	0.154	0.234	0.119	0.189
	CR	0.936	0.960	0.934	0.960	0.940	0.944
(400,20%,30%)	Bias	-0.026	0.007	-0.025	0.007	-0.002	-0.001
	ESE	0.118	0.169	0.117	0.169	0.087	0.128
	SEE	0.109	0.160	0.109	0.160	0.085	0.133
	CR	0.934	0.970	0.938	0.970	0.946	0.964
(600,20%,30%)	Bias	-0.025	0.020	-0.023	0.020	-0.002	0.010
	ESE	0.096	0.144	0.095	0.145	0.069	0.106
	SEE	0.089	0.131	0.089	0.131	0.069	0.108
	CR	0.936	0.940	0.934	0.938	0.944	0.960
(1000,20%,30%)	Bias	-0.019	0.008	-0.017	0.010	-0.002	0.005
	ESE	0.077	0.099	0.074	0.098	0.052	0.075
	SEE	0.072	0.101	0.069	0.099	0.054	0.084
	CR	0.942	0.956	0.946	0.952	0.946	0.970
(200,40%,50%)	Bias	-0.037	0.023	-0.036	0.022	-0.001	0.016
	ESE	0.177	0.273	0.176	0.273	0.139	0.217
	SEE	0.169	0.250	0.169	0.250	0.142	0.214
	CR	0.940	0.966	0.942	0.966	0.954	0.958
(400,40%,50%)	Bias	-0.032	0.021	-0.029	0.020	-0.002	0.009
	ESE	0.124	0.172	0.119	0.171	0.099	0.152
	SEE	0.119	0.169	0.119	0.169	0.100	0.150
	CR	0.940	0.952	0.950	0.950	0.946	0.948
(600,40%,50%)	Bias	-0.030	0.024	-0.029	0.024	-0.003	0.007
	ESE	0.117	0.159	0.109	0.159	0.081	0.123
	SEE	0.103	0.145	0.103	0.144	0.082	0.123
	CR	0.945	0.958	0.945	0.960	0.950	0.966
(1000,40%,50%)	Bias	-0.025	0.007	-0.021	0.009	0.005	-0.009
	ESE	0.077	0.108	0.074	0.105	0.058	0.095
	SEE	0.077	0.109	0.075	0.106	0.063	0.095
	CR	0.946	0.962	0.952	0.958	0.970	0.960

Bias: bias of parameter estimator; ESE: the empirical standard error; SEE: average of the standard error estimator; CR: coverage rate of the 95% confidence interval; Cure: Cure rate; Cen.: Censoring rate.

Table 4: Simulation summaries for the proposed estimators of the *short-term* effect under PHPH model.

(n,Cure,Cen.)		PO				MLE	
		$\hat{\phi}^{\mathrm{PO}}_{1,\mathrm{NP}}$	$\hat{\phi}^{\mathrm{PO}}_{2,\mathrm{NP}}$	$\hat{\phi}^{\mathrm{PO}}_{1,\mathrm{KM}}$	$\hat{\phi}^{\mathrm{PO}}_{2,\mathrm{KM}}$	$\hat{\phi}^{\mathrm{MLE}}_{1}$	$\hat{\phi}^{\mathrm{MLE}}_{2}$
(200,20%,30%)	Bias	0.021	0.003	0.018	0.003	0.007	-0.002
	ESE	0.355	0.175	0.348	0.175	0.194	0.114
	SEE	0.327	0.171	0.325	0.170	0.189	0.113
	CR	0.950	0.948	0.952	0.948	0.950	0.954
(400,20%,30%)	Bias	-0.002	0.000	-0.002	0.000	0.004	-0.002
	ESE	0.260	0.147	0.260	0.147	0.132	0.079
	SEE	0.233	0.129	0.233	0.129	0.132	0.079
	CR	0.954	0.940	0.954	0.940	0.950	0.956
(600,20%,30%)	Bias	-0.028	0.007	-0.028	0.008	-0.012	0.001
	ESE	0.225	0.104	0.225	0.104	0.113	0.066
	SEE	0.192	0.102	0.192	0.103	0.108	0.064
	CR	0.946	0.936	0.944	0.934	0.944	0.948
(1000,20%,30%)	Bias	0.000	0.005	-0.004	0.009	-0.002	0.000
	ESE	0.142	0.073	0.140	0.079	0.083	0.049
	SEE	0.141	0.073	0.140	0.079	0.083	0.049
	CR	0.954	0.952	0.956	0.954	0.938	0.956
(200,40%,50%)	Bias	-0.010	0.003	-0.010	0.003	-0.005	-0.002
	ESE	0.398	0.231	0.398	0.231	0.213	0.142
	SEE	0.359	0.196	0.360	0.196	0.209	0.136
	CR	0.938	0.926	0.938	0.925	0.964	0.942
(400,40%,50%)	Bias	-0.009	0.018	-0.008	0.017	0.001	0.005
	ESE	0.268	0.154	0.269	0.154	0.147	0.103
	SEE	0.255	0.139	0.255	0.139	0.146	0.095
	CR	0.950	0.924	0.952	0.922	0.950	0.920
(600,40%,50%)	Bias	-0.034	0.014	-0.036	0.015	-0.006	0.002
	ESE	0.228	0.141	0.229	0.140	0.126	0.081
	SEE	0.225	0.122	0.226	0.123	0.119	0.077
	CR	0.944	0.930	0.945	0.930	0.932	0.938
(1000,40%,50%)	Bias	-0.020	0.017	-0.021	0.021	-0.002	-0.001
	ESE	0.146	0.083	0.142	0.081	0.088	0.065
	SEE	0.149	0.083	0.146	0.081	0.091	0.059
	CR	0.940	0.945	0.940	0.945	0.968	0.944

Bias: bias of parameter estimator; ESE: the empirical standard error; SEE: average of the standard error estimator; CR: coverage rate of the 95% confidence interval; Cure: Cure rate; Cen.: Censoring rate.

Table 5: Simulation summaries for variable selection on the *incidence* and *latency* component of the PHMC model.

		40% censoring rate			60% censoring rate		
n		MSE	TP	FP	MSE	TP	FP
				**Incidence**			
400	Full.NP	2.87	-	-	6.14	-	-
	Full.KM	2.91	-	-	6.28	-	-
	Oracle.NP	0.75	4	0	3.75	4	0
	Oracle.KM	0.75	4	0	3.54	4	0
	SCAD.NP	1.83	2.95	0.66	4.80	0.56	0.44
	SCAD.KM	1.83	3.02	0.65	4.80	0.57	0.41
	LASSO	1.81	3.16	1.88	4.37	0.63	0.61
	ALASSO	1.77	3.16	1.60	4.41	0.54	0.45
1000	Full.NP	0.77	-	-	4.18	-	-
	Full.KM	0.71	-	-	4.17	-	-
	Oracle.NP	0.28	4	0	3.13	4	0
	Oracle.KM	0.27	4	0	3.05	4	0
	SCAD.NP	0.44	3.93	0.28	3.91	1.26	0.74
	SCAD.KM	0.44	3.93	0.30	3.91	1.29	0.77
	LASSO	0.78	3.94	2.37	3.97	1.44	1.56
	ALASSO	0.76	3.94	2.03	3.79	1.88	1.34
				**Latency**			
400	Full.indep	0.62	-	-	1.78	-	-
	Full.exch	0.76	-	-	2.55	-	-
	Full.ar1	0.66	-	-	2.06	-	-
	Oracle.indep	0.19	4	0	0.65	4	0
	Oracle.exch	0.20	4	0	0.83	4	0
	Oracle.ar1	0.18	4	0	0.70	4	0
	SCAD.indep	0.36	3.79	1.47	1.69	1.78	1.98
	SCAD.exch	0.40	3.69	0.47	1.95	1.29	0.93
	SCAD.ar1	0.42	3.69	0.41	1.85	1.23	0.44
	LASSO	0.45	3.84	3.07	1.95	1.16	0.79
	ALASSO	0.23	3.92	0.57	1.08	2.38	0.49
1000	Full.indep	0.21	-	-	1.06	-	-
	Full.exch	0.23	-	-	1.21	-	-
	Full.ar1	0.22	-	-	1.24	-	-
	Oracle.indep	0.07	4	0	0.51	4	0
	Oracle.exch	0.08	4	0	0.61	4	0
	Oracle.ar1	0.07	4	0	0.54	4	0
	SCAD.indep	0.13	3.94	0.34	0.91	3.07	1.31
	SCAD.exch	0.22	3.88	0.21	1.19	2.18	0.48
	SCAD.ar1	0.18	3.87	0.13	0.93	1.92	0.24
	LASSO	0.19	3.99	2.11	0.96	3.41	2.17
	ALASSO	0.07	3.99	0.14	0.44	3.81	0.29

Full: model includes all covariates; Oracle: model only includes the covariates with nonzero coefficients; Acronyms that ends with .NP or .KM indicates pseudo-observations $\hat{\pi}^i_{\mathrm{NP}}$ or $\hat{\pi}^i_{\mathrm{KM}}$; Acronyms that ends with .indep, .exch and .ar1 indicates independence, exchangeable and AR(1) correlation structure among pseudo-observations; LASSO: PHMC model with LASSO penalty; ALASSO: PHMC model with ALASSO penalty; MSE: average estimated mean square error; TP: the average true positives; FP: the average false positives.

Table 6: Simulation summaries for variable selection of the *long-term* effect under PHPH model based on 200 replications.

		40% censoring rate						60% censoring rate					
		$\hat{\theta}^i_{\text{NP}}$			$\hat{\theta}^i_{\text{KM}}$			$\hat{\theta}^i_{\text{NP}}$			$\hat{\theta}^i_{\text{KM}}$		
n		MSE	TP	FP	MSE	TP	FP	MSE	TP	FP	MSE	TP	FP
200	Full	4.99	-	-	4.82	-	-	7.38	-	-	7.37	-	-
	Oracle	1.24	3	0	1.24	3	0	2.16	3	0	2.15	3	0
	SCAD.indep	2.09	2.28	2.88	2.09	2.28	2.89	3.29	1.32	1.81	3.32	1.31	1.82
400	Full	3.32	-	-	3.29	-	-	5.11	-	-	5.09	-	-
	Oracle	1.19	3	0	1.19	3	0	2.39	3	0	2.41	3	0
	SCAD.indep	1.91	2.63	2.79	1.92	2.63	2.81	3.15	1.60	1.78	3.16	1.59	1.69
600	Full	1.89	-	-	1.88	-	-	4.53	-	-	4.69	-	-
	Oracle	1.09	3	0	1.09	3	0	2.09	3	0	2.09	3	0
	SCAD.indep	1.63	2.68	2.17	1.63	2.69	2.16	3.06	1.67	1.50	3.07	1.67	1.47
1000	Full	1.70	-	-	1.71	-	-	2.78	-	-	2.81	-	-
	Oracle	0.90	3	0	0.90	3	0	1.86	3	0	1.86	3	0
	SCAD.indep	1.58	2.83	1.47	1.58	2.84	1.53	2.80	1.75	1.19	2.81	1.76	1.22

Full: model includes all covariates; Oracle: model only includes the covariates with nonzero coefficients; SCAD.indep: the proposed PGEE with independence structure among pseudo-observations; MSE: the average estimated mean square error; TP: the average true positives; FP: the average false positives.

Table 7: Simulation summaries for variable selection of the *short-term* effect under PHPH model based on 200 replications.

		40% censoring rate						60% censoring rate					
		$\hat{F}^i_{\mathrm{NP}}(\boldsymbol{t})$			$\hat{F}^i_{\mathrm{KM}}(\boldsymbol{t})$			$\hat{F}^i_{\mathrm{NP}}(\boldsymbol{t})$			$\hat{F}^i_{\mathrm{KM}}(\boldsymbol{t})$		
n		MSE	TP	FP	MSE	TP	FP	MSE	TP	FP	MSE	TP	FP
200	Full.indep	2.51	-	-	2.50	-	-	5.49	-	-	5.58	-	-
	Full.exch	4.40	-	-	4.38	-	-	7.24	-	-	7.68	-	-
	Full.ar1	3.67	-	-	3.65	-	-	6.29	-	-	5.89	-	-
	Oracle.indep	0.64	3	0	0.64	3	0	1.80	3	0	1.81	3	0
	Oracle.exch	0.83	3	0	0.83	3	0	2.48	3	0	2.43	3	0
	Oracle.ar1	1.01	3	0	0.73	3	0	2.31	3	0	2.31	3	0
	SCAD.indep	1.48	1.07	1.56	1.41	1.06	1.55	1.84	0.26	0.97	1.91	0.26	0.96
	SCAD.exch	1.52	0.70	0.71	1.51	0.69	0.66	1.85	0.20	0.78	1.98	0.19	0.68
	SCAD.ar1	1.46	0.69	0.45	1.42	0.69	0.50	1.86	0.12	0.28	1.74	0.10	0.23
400	Full.indep	1.15	-	-	1.16	-	-	2.57	-	-	2.73	-	-
	Full.exch	1.68	-	-	1.63	-	-	4.41	-	-	4.83	-	-
	Full.ar1	1.34	-	-	1.35	-	-	3.45	-	-	3.47	-	-
	Oracle.indep	0.40	3	0	0.40	3	0	1.16	3	0	1.18	3	0
	Oracle.exch	0.51	3	0	0.51	3	0	1.42	3	0	1.45	3	0
	Oracle.ar1	0.72	3	0	0.45	3	0	1.15	3	0	1.24	3	0
	SCAD.indep	0.85	1.81	2.22	0.84	1.81	2.27	1.42	0.40	0.87	1.41	0.41	0.86
	SCAD.exch	1.00	1.61	0.93	1.01	1.58	0.82	1.55	0.24	0.45	1.58	0.25	0.61
	SCAD.ar1	0.92	1.59	0.62	0.92	1.58	0.67	1.51	0.16	0.14	1.47	0.18	0.22
600	Full.indep	0.77	-	-	0.77	-	-	1.98	-	-	1.98	-	-
	Full.exch	1.03	-	-	1.04	-	-	2.97	-	-	3.06	-	-
	Full.ar1	0.87	-	-	0.88	-	-	2.21	-	-	2.24	-	-
	Oracle.indep	0.33	3	0	0.33	3	0	1.00	3	0	1.00	3	0
	Oracle.exch	0.41	3	0	0.41	3	0	1.23	3	0	1.27	3	0
	Oracle.ar1	0.60	3	0	0.37	3	0	0.98	3	0	1.05	3	0
	SCAD.indep	0.60	2.05	1.85	0.59	2.08	1.90	1.32	0.60	1.15	1.37	0.64	1.24
	SCAD.exch	0.77	1.90	0.47	0.75	1.93	0.46	1.47	0.33	0.63	1.46	0.38	0.75
	SCAD.ar1	0.67	1.90	0.38	0.66	1.94	0.43	1.40	0.25	0.23	1.38	0.26	0.24
1000	Full.indep	0.51	-	-	0.51	-	-	1.38	-	-	1.35	-	-
	Full.exch	0.69	-	-	0.66	-	-	1.85	-	-	1.97	-	-
	Full.ar1	0.57	-	-	0.58	-	-	1.50	-	-	1.51	-	-
	Oracle.indep	0.24	3	0	0.24	3	0	0.83	3	0	0.82	3	0
	Oracle.exch	0.28	3	0	0.27	3	0	0.97	3	0	0.98	3	0
	Oracle.ar1	0.26	3	0	0.26	3	0	0.78	3	0	0.89	3	0
	SCAD.indep	0.49	2.14	1.18	0.49	2.12	1.28	1.23	0.81	1.26	1.23	0.83	1.34
	SCAD.exch	0.56	2.09	0.32	0.52	2.11	0.30	1.38	0.50	0.60	1.41	0.52	0.65
	SCAD.ar1	0.53	2.07	0.18	0.52	2.03	0.17	1.32	0.37	0.20	1.37	0.35	0.21

Full: model includes all covariates ;Oracle: model only includes the covariates with nonzero coefficients; SCAD: the proposed PGEE with SCAD penality funcrion; Acronyms that ends with .indep, .exch and .ar1 indicates independence, exchangeable and AR(1) correlation structure among pseudo-observations; MSE: the average estimated mean square error; TP: the average true positives; FP: the average false positives.

Table 8: Cure rate estimates for the melanoma data.

**Group**	$\hat{S}_{\text{KM}}(t_{max})$	$\hat{\boldsymbol{\alpha}}^{\text{EM}}$	$\hat{\boldsymbol{\alpha}}^{\text{PO}}_{\text{NP}}$	$\hat{\boldsymbol{\alpha}}^{\text{PO}}_{\text{KM}}$	$\hat{\boldsymbol{\gamma}}^{\text{PO}}_{\text{NP}}$	$\hat{\boldsymbol{\gamma}}^{\text{PO}}_{\text{KM}}$
Treatment/Male	37.2%	34.0%	39.4%	38.4%	42.4%	41.0%
Treatment/Female	33.8%	30.5%	34.4%	33.3%	36.1%	34.8%
Control/Male	15.2%	18.2%	15.7%	14.9%	18.5%	17.9%
Control/Female	28.6%	26.2%	26.4%	25.4%	28.1%	27.2%

KM: Kaplan-Meier estimator

Web Appendix F

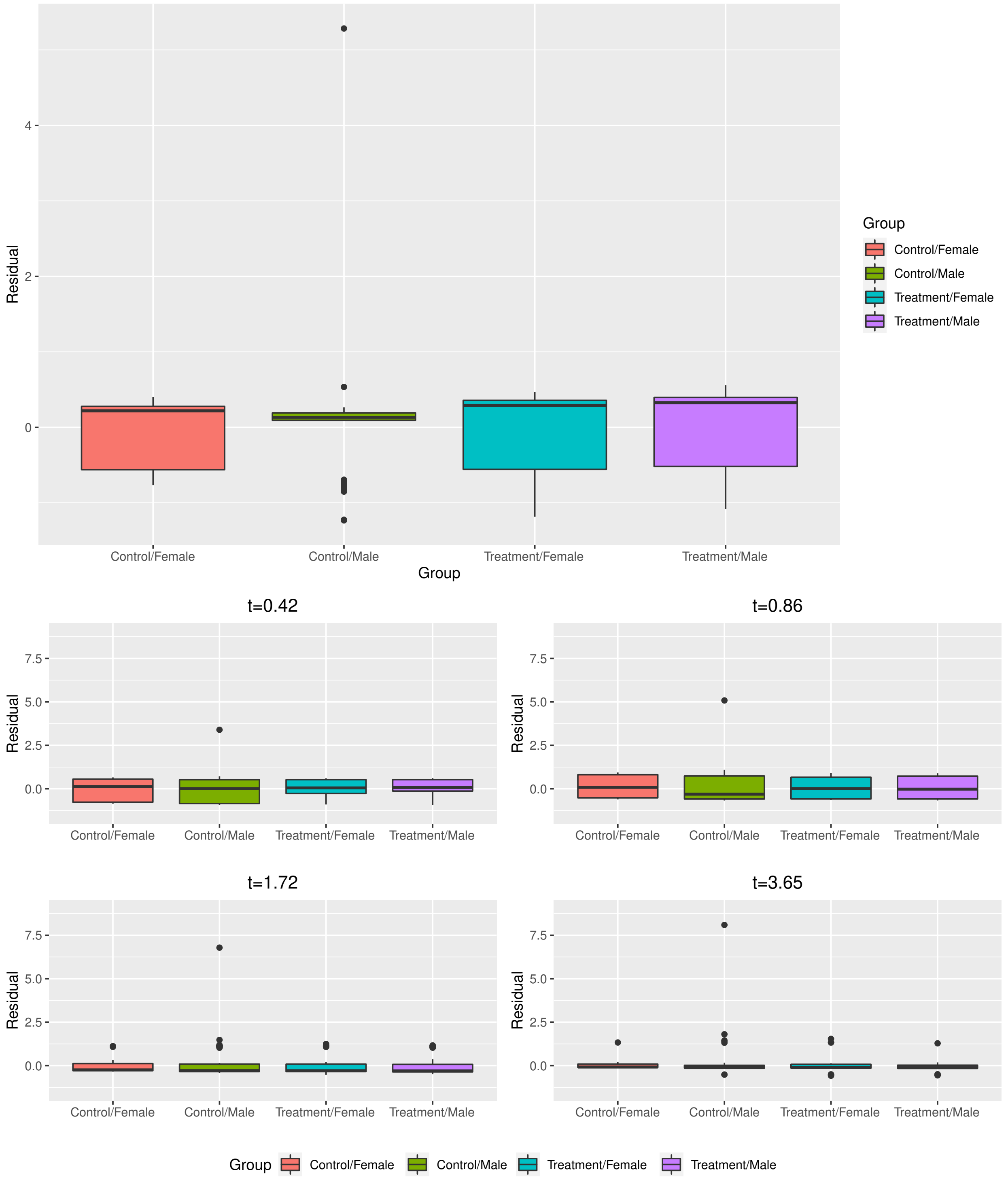

Figure 2: Boxplots of pseudo-residuals under the PHMC model for the melanoma data. The top panel shows the boxplots of pseudo-residuals stratified by treatment and gender based on the pseudo-observations $\hat{\pi}^i_{\mathrm{KM}}$ in (3.6) of the main manuscript. The bottom panel presents the boxplots of pseudo-residuals based on pseudo-observations $\hat{S}^i_u(t)$ in (3.8) of the main manuscript calculated at four given time points chosen from the quantiles of observed event times.

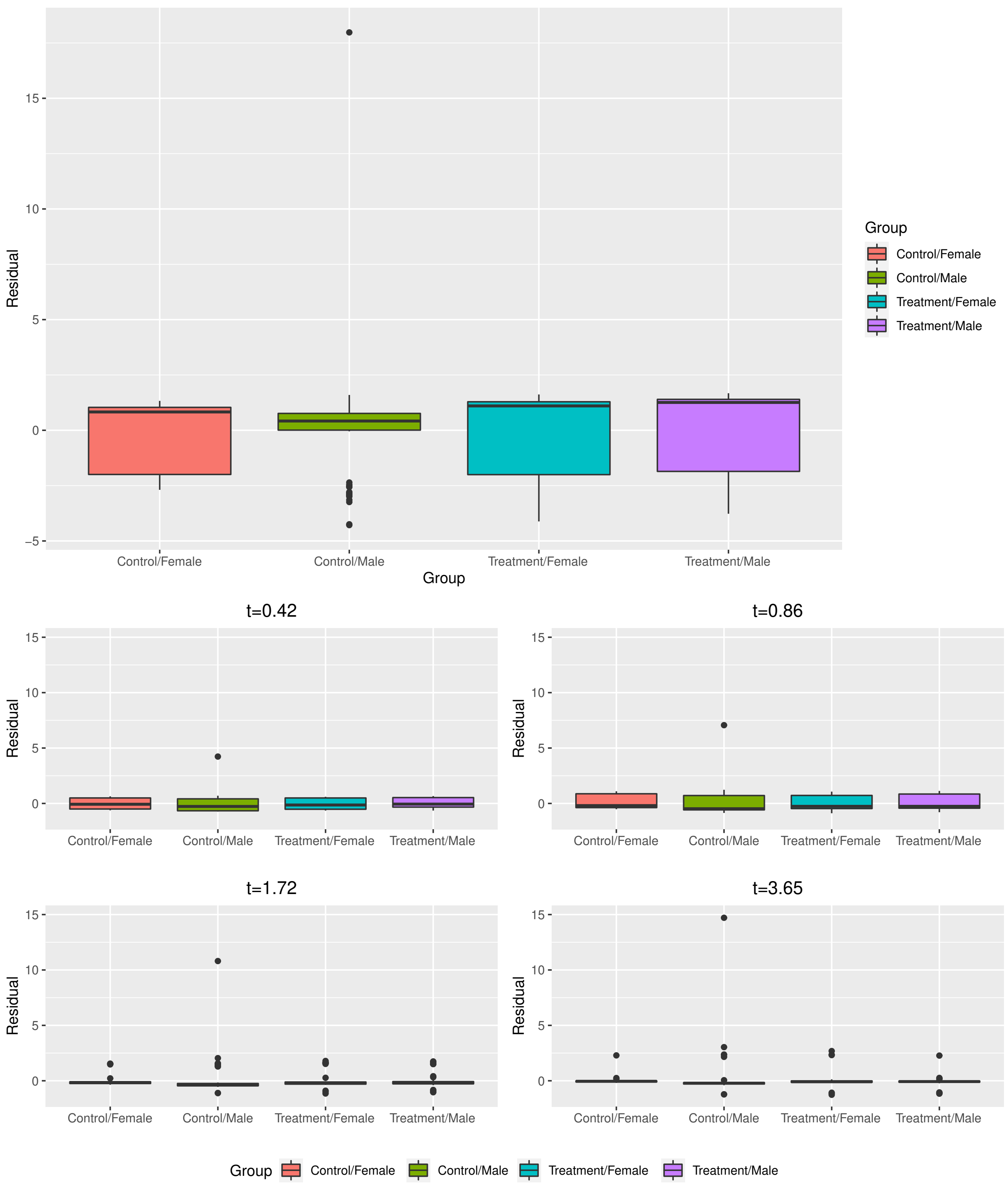

Figure 3: Boxplots of pseudo-residuals under the PHPH model for the melanoma data. The top panel shows the boxplots of pseudo-residuals stratified by treatment and gender based on the pseudo-observations $\hat{\theta}^i_{\mathrm{KM}}$ in (3.9) of the main manuscript. The bottom panel presents the boxplots of pseudo-residuals based on pseudo-observations $\hat{F}^i_{\mathrm{KM}}(t)$ in (3.11) of the main manuscript calculated at four given time points.

References

1. Tsodikov, A. (2001). Estimation of survival based on proportional hazards when cure is a possibility. *Mathematical and Computer modelling*, **33**(12-13), 1227-1236.

2. Jacobsen, M., & Martinussen, T. (2016). A note on the large sample properties of estimators based on generalized linear models for correlated pseudo-observations. *Scandinavian Journal of Statistics*, **43**(3), 845-862.

3. Overgaard, M., Parner, E. T., & Pedersen, J. (2017). Asymptotic theory of generalized estimating equations based on jack-knife pseudo-observations. *Annals of Statistics*, **45**(5), 1988-2015.

4. Peng Y. and Dear K. A nonparametric mixture model for cure rate estimation. *Biometrics* 2000; **56**(1): 237–243.

5. Cai C., Zou Y., Peng Y. et al. smcure: An R-package for estimating semiparametric mixture cure models. *Comput Methods Programs Biomed* 2012; **108**(3): 1255–1260.
